# Multi-echo acquisition and thermal denoising advances precision functional imaging

**DOI:** 10.1162/imag_a_00426

**Published:** 2025-01-09

**Authors:** Julia Moser, Steven M. Nelson, Sanju Koirala, Thomas J. Madison, Alyssa K. Labonte, Cristian Morales Carrasco, Eric Feczko, Lucille A. Moore, Jacob T. Lundquist, Kimberly B. Weldon, Gracie Grimsrud, Kristina Hufnagle, Weli Ahmed, Michael J. Myers, Babatunde Adeyemo, Abraham Z. Snyder, Evan M. Gordon, Nico U.F. Dosenbach, Brenden Tervo-Clemmens, Bart Larsen, Steen Moeller, Essa Yacoub, Luca Vizioli, Kamil Uğurbil, Timothy O. Laumann, Chad M. Sylvester, Damien A. Fair

**Affiliations:** Masonic Institute for the Developing Brain, University of Minnesota, Minneapolis, MN, United States; Department of Pediatrics, University of Minnesota, Minneapolis, MN, United States; Institute of Child Development, University of Minnesota, Minneapolis, MN, United States; Department of Psychiatry, Washington University in St. Louis, St. Louis, MO, United States; Department of Neurology, Washington University School of Medicine, St Louis, MO, United States; Department of Radiology, Washington University in St. Louis, St. Louis, MO, United States; Department of Psychological and Brain Sciences, Washington University in St. Louis, St Louis, MO, United States; Department of Pediatrics, Washington University School of Medicine, St Louis, MO, United States; Department of Biomedical Engineering, Washington University in St. Louis, St Louis, MO, United States; Center for Magnetic Resonance Research (CMRR), University of Minnesota, Minneapolis, MN, United States; Taylor Family Institute for Innovative Research, Washington University in St. Louis, St. Louis, MO, United States

**Keywords:** developmental neuroimaging, precision functional mapping, multi-echo, neonates, brain development, methodological advancements

## Abstract

The characterization of individual functional brain organization with Precision Functional Mapping has provided important insights in recent years in adults. However, little is known about the ontogeny of inter-individual differences in brain functional organization during human development. Precise characterization of systems organization during periods of high plasticity is likely to be essential for discoveries promoting lifelong health. Obtaining precision functional magnetic resonance imaging (fMRI) data during development has unique challenges that highlight the importance of establishing new methods to improve data acquisition, processing, and analysis. Here, we investigate two methods that can facilitate attaining this goal: multi-echo (ME) data acquisition and thermal noise removal with Noise Reduction with Distribution Corrected (NORDIC) principal component analysis. We applied these methods to precision fMRI data from adults, children, and newborn infants. In adults, both ME acquisitions and NORDIC increased temporal signal to noise ratio (tSNR) as well as the split-half reliability of functional connectivity matrices, with the combination helping more than either technique alone. The benefits of NORDIC denoising replicated in both our developmental samples. ME acquisitions revealed longer and more variable T2* relaxation times across the brain in infants relative to older children and adults, leading to major differences in the echo weighting for optimally combining ME data. This result suggests ME acquisitions may be a promising tool for optimizing developmental fMRI, albeit application in infants needs further investigation. The present work showcases methodological advances that improve Precision Functional Mapping in adults and developmental populations and, at the same time, highlights the need for further improvements in infant-specific fMRI.

## 1 Introduction

Brain regions that are functionally connected can be identified by correlation analysis of blood oxygen level dependent (BOLD) signals—the endogenous contrast used for functional magnetic resonance imaging (fMRI;Biswal et al., 1995;Ogawa et al., 1990). Although common patterns of functional brain organization have been identified via group average studies of functional connectivity among brain regions e.g., (Gordon et al., 2016;Power et al., 2011;Yeo et al., 2011), considerable inter-individual variability exists around these global patterns (Gordon et al., 2017). Under the right conditions, these patterns are fairly stable within individuals (Gratton et al., 2018;Laumann et al., 2015). Precision Functional Mapping (PFM) is a technique that allows for a reliable characterization of such person-specific functional systems (i.e., network maps or brain topography, and connection strengths or brain topology), traditionally made possible by acquiring abundant data (i.e., several hours) across multiple imaging sessions of the same participant (Gordon et al., 2017).

### 1.1 Precision Functional Mapping in developmental neuroimaging

The ability to reliably and precisely map functional systems in individuals has provided an important platform for a whole host of discoveries in recent years in adults. Insights from PFM, for example, have led to a rethinking of the classic homunculus (Penfield & Boldrey, 1937) detecting inter-effector regions that interrupt effector-specific areas, forming the somato-cognitive action network (Gordon et al., 2023) and expanded our understanding of experience dependent plasticity (Newbold et al., 2020). Furthermore, recent evidence has revealed distinct patterns of expansion of the salience network in individuals with major depression (Lynch et al., 2023), demonstrating that PFM can provide important information for designing targeted neuromodulation treatments (Elbau et al., 2023). Functional brain organization in adults is as personalized as a fingerprint (Miranda-Dominguez et al., 2014) with areas of high and low probability of network overlap between persons (Hermosillo et al., 2024). Even during childhood and adolescence, personalized network topography is providing insights into the associations between brain development, cognition, age (Cui et al., 2020), and psychopathology (Cui et al., 2022). However, despite these accumulating discoveries, little is known about the ontogeny of individual differences in brain functional organization during the earliest periods of life, when individual-specific brain organization may be most informative about lifelong developmental trajectories.

The commonly used strategy of acquiring large amounts of data across multiple sessions for PFM has major limitations in many populations. For example, gathering large amounts of fMRI data in newborns and infants requires research teams to adopt specialized data acquisition strategies (Dubois et al., 2021;Korom et al., 2021) not necessarily amenable to repeated sessions. In addition, in most cases, data are acquired during natural sleep, when spontaneous waking can limit the acquisition of large amounts of, motion-free data. These data acquisition challenges are accompanied by additional inherent technical challenges of infant imaging, including the lack of commercially available head coils optimized for developmental populations, resulting in sub-optimal signal to noise ratio (SNR) when using adult coils (Dubois et al., 2021). SNR can be improved by spatially smoothing or averaging data across parcels. However, these strategies lead to decreased spatial precision, which is the crux of personalized PFM. Even where large amounts of data can be collected across multiple sessions, PFM in developmental populations is still limited simply by the fast pace at which the brain develops, particularly during early stages of maturation. The rapid growth rate of the infant brain requires collecting multiple datasets in a much shorter time-period compared to a child or an adult sample for which multiple sessions of low-motion data can be collected over the course of weeks to months. Taken together, studies investigating individual differences in functional brain architecture require data that are both spatially precise and reliable to generate personalized functional brain maps, which are features that are particularly challenging to achieve in developmental samples.

### 1.2 These challenges call for improvements in data collection and processing methods

These challenges highlight the importance of identifying data acquisition, processing, and analysis strategies that maximize signal and reduce the time in the scanner needed to obtain reliable characterization of functional network topographies and their underlying topology. This paper examines two possible methodological improvements for developmental neuroimaging that previously and independently have been shown to increase signal quality and reliability in adult participants: 1) Multi-echo (ME) data acquisition (Lynch et al., 2020) and 2) Noise Reduction with Distribution Corrected (NORDIC) principal component analysis (Dowdle et al., 2023;Moeller et al., 2021;Vizioli et al., 2021).

In contrast to traditional BOLD imaging, ME data acquisitions capture images at multiple echo times during a single readout time of the T2*, or transverse relaxation signal decay. The T2* weighted fMRI signal reflects the decay in transverse magnetization introduced by the radio frequency (RF) pulse. Following the RF pulse, the fMRI signal decays exponentially over successive echoes (Posse, 2012; see Fig. 5A for an example). T2* relaxation times vary across age, brain regions, and tissue types, due to differences in underlying neurobiological tissue properties. The optimal echo time to capture an image (i.e., tradeoff between signal to noise and functional contrast) is usually defaulted to the T2* relaxation time of the voxels of interest. Voxels with longer T2*s have higher signal intensities in images from longer echo times compared to voxels with shorter T2*s (Kundu et al., 2017). With ME data acquisition, data from different echo times can be optimally combined based on the T2* of the underlying tissue (Posse et al., 1999). The properties of the signal decay across echoes can furthermore be used to separate BOLD effects from non-BOLD effects using multi-echo independent component analysis (MEICA;Kundu et al., 2012).

The technique of ME data acquisition has been known for some time (e.g.,Posse et al., 1999) but has struggled to gain popularity owing to the requisite compromises in spatial and temporal resolution as well as the need to use in-plane accelerations to avoid excessively long echo trains during image readout. NORDIC, a recently developed tool for denoising fMRI data, could help to overcome some of these challenges and further improve the capabilities and usability of ME data. NORDIC enhances image quality by removing zero-mean, unstructured thermal noise, thereby improving the SNR without sacrificing spatial precision (Dowdle et al., 2023;Vizioli et al., 2021). However, little is known about the value of ME-NORDIC fMRI, particularly in pediatric populations.

### 1.3 Translating methods from adults to infants—challenges and opportunities

Most methodological advances are first established in healthy young adults. However, their transferability to developmental neuroimaging is not guaranteed. Particularly for very young populations, it is important to consider that infant brains are not merely smaller adult brains but instead have specific properties that change with their developmental stage. T2* relaxation, which forms the basis of fMRI, shows a developmental trend, being slower in newborns compared to infants and adults (Rivkin et al., 2004;Williams et al., 2005). This slower T2* decay is related to several factors, including reduction in the amount of free water compared to bound water in brain tissues over development (Engelbrecht et al., 1998;Xydis et al., 2006) as axon myelination increases in both white and gray matter (Baumann & Pham-Dinh, 2001;Dubois et al., 2014;Kostović et al., 2019). Other factors are an increase in proton density, an abundance of macromolecules, and changes in iron concentration (Goksan et al., 2017). These are important factors to consider for the evaluation of a prospective ME acquisition in this age group as the optimal combination of echoes is dependent on the T2* relaxation time of a given voxel. Given the impact of brain developmental changes on T2* relaxation times, ME data acquisitions could, indeed, be a promising tool for developmental imaging. It opens up the possibility to account for the impact of brain developmental differences on fMRI signals. This may have particular benefit for longitudinal investigations, in which individually optimized echo combinations based on T2* times could be determined for each acquisition time point while still using the same acquisition sequence.

### 1.4 Methods under investigation in the present study

The present study is an individualized study in which we investigated T2* relaxation times and optimal echo combinations of ME fMRI data across different ages and the impact of NORDIC thermal noise removal on these data. To test the usability and benefit of these methodological approaches, we utilized precision imaging data with single-echo (SE) and ME data acquisitions from adults, children, and newborns acquired across multiple days. We compared ME infant and adult data acquired with the same sequence to gain a better understanding of ME data acquisition and optimal echo times in infants compared to adults, for whom ME fMRI had previously been shown to provide advantages over SE acquisitions (Lynch et al., 2020). MEICA is not included in the present investigation as its ability to separate BOLD effects from non-BOLD effects is based on signal decay curve shapes, which are different in young infants and not sufficiently understood yet. We used NORDIC denoising on all datasets to look at differences in data quality with and without thermal noise removal. We hypothesize that the application of NORDIC denoising, with its targeting of thermal noise, will be useful independent of the age of the target sample. Data quality was evaluated using temporal SNR (tSNR), strength of functional connections, and split-half reliability of brain functional connectivity within individuals. Even though tSNR is not comparable between datasets with different acquisition sequences (Triantafyllou et al., 2006), its usage contributes to solving the question whether effects of NORDIC and ME optimal echo combination are additive or multiplicative or redundant. This investigation informs the application of ME and NORDIC to developmental neuroimaging with the goal of improving signal quality to facilitate precision functional imaging.

## 2 Methods

### 2.1 Sample

The sample consists of one adult (PA001) and two children (PC001 and PC002; age 10) enrolled at the University of Minnesota (UMN), and one adult (PA002) and three healthy neonates enrolled at Washington University in St. Louis (WashU), ages 28 days (43 weeks postmenstrual age (wPMA); PB004), 12 days (41 wPMA; PB005), and 13 days (41 wPMA; PB001). Data included in this manuscript were collected for studies approved by the UMN’s Institutional Review Board and the Human Studies Committees at WashU, and written informed consent was obtained from all participants and parents of minor participants. For PA001, ME and SE data were acquired in four sessions over 3 months. For PA002 ME, data were acquired in six sessions over 4 months and SE data were acquired in three sessions over 1 month. For PC001 and PC002, data were acquired in four sessions over 8 months, and for neonatal participants, data were acquired over 4 to 5 days within 1 week. Anatomical and resting-state data in neonates were acquired during natural sleep.

### 2.2 Data acquisition

rs-fMRI data for all subjects (adults, children, and infants) were acquired with the CMRR multiband (MB)-ME sequence (Feinberg et al., 2010;Moeller et al., 2010) with 5 echoes at WashU and 4 echoes at UMN—dropping the last echo based on compatibility issues with Siemens XA30 (14.2, 38.93, 63.66, 88.39, 113.12 ms; TR = 1.761 s, 2 mm resolution, MB factor = 6, IPAT = 2, flip angle = 68°). Data were acquired in PA phase encoding direction. The total amount of rs-fMRI data acquired ranged between 72 min (PB004) and 203 min (PA002; seeSupplementary Table 1). Additionally, spin echo fieldmaps were acquired in both AP and PA direction (SE, single-band, 3 frames per run, TR = 8.0 s, TE = 66 ms, flip angle = 90°) for all participants.

We additionally acquired single-echo rs-fMRI data for adult subjects and infants PB004 and PB005 with a more standard fMRI acquisition protocol (adults: TR 0.8 s, TE = 37 ms, 2 mm resolution, MB factor = 8, flip angle = 52°; infants: TR 1.51 s, TE = 37 ms, 2 mm resolution, MB factor = 4, flip angle = 52°, PA phase encoding direction; see alsoSupplementary Table 2). The total amount of SE rs-fMRI data acquired ranged between 77 min (PB004) and 159 min (PA001; seeSupplementary Table 1).

Imaging for PA001 and PC subjects was performed at UMN using a Siemens 3-T Prisma scanner and a 64 channel (PC001) and a 32 channel (PA001, PC002) head coil. At WashU, functional imaging for PA002 was performed using a Siemens 3-T Prisma scanner and a 64 channel head coil. Neonatal imaging was performed using a Siemens 3-T Prisma scanner and a 32 channel head coil. For details on anatomical references, seeSupplemental Methods.

### 2.3 Data processing

Data for all processing and analysis were converted to BIDS format with dcm2bids (Boré et al., 2023). For analyses in which NORDIC was applied to a dataset, this step was performed before the regular preprocessing using the phase and the magnitude images of the scan data. Using phase and magnitude images for NORDIC allows to maintain complex-valued Gaussian noise (seeMoeller et al., 2021for details). Three noise frames acquired at the end of each functional run were used to help estimate the empirical thermal noise level. For the one participant in which noise scans were not acquired (PB001), we used a theoretical thermal noise level (implemented as 1/sqrt(2);Moeller et al., 2021;Vizioli et al., 2021). NORDIC was implemented in Matlab R2019a and applied to data from each run (Vizioli et al., 2021). In the case of ME data acquisition, NORDIC denoising was performed for each echo individually.

Adult and children’s imaging data were processed using a recently upgraded version of fMRIprep (Esteban et al. (2019); version 24.0.0, development version dev55 + g4d21c37a; github commit 4d21c37a), now consistent with processes outlined for ABCD-BIDS (Sturgeon, Earl, et al., 2023). Susceptibility distortion correction of BOLD time series was done within fMRIPrep, using an FSL topup-based method to estimate fieldmaps from “PEPolar” acquisitions (acquisitions with opposite phase encoding direction;Andersson et al., 2003). These fieldmaps were then used to correct distortion of the BOLD time series data. Additional options enabled for fMRIPrep processing were “—project-goodvoxels”, to exclude voxels with locally high coefficient of variation from volume-to-surface projection of the BOLD time series, and “—cifti-output 91k” to enable output of BOLD time series and morphometric data (surface curvature, sulcal depth, and cortical thickness maps) in the HCP grayordinates space (Glasser et al., 2013). This updated version of fMRIprep optimally combines ME data within its processing workflow using T2* based echo weighting ($w_{TE}=TE*e^{\frac{-TE}{T2*}}$;Posse et al., 1999).

After fMRIPrep processing, functional connectivity postprocessing was performed using XCP-D 0.6.1 (Mehta et al., 2023) with options “—cifti” (ingress CIFTI fMRIPrep CIFTI derivatives), “—warp-surfaces-native2std” (apply the T1w-atlas nonlinear transform from the fMRIPrep derivatives to align XCP-D’s surface mesh output to the MNI152NLin6Asym template), “-m” (after processing, concatenate BOLD timeseries and motion data across runs), “—dcan-qc” (generate a QC report in similar format to the “executive summary” of the DCAN ABCD-BIDS pipeline), “—despike” (apply AFNI 3dDespike to BOLD timeseries data), “—lower-bpf” 0.009 (set lower bound of band-pass filter to 0.009 Hz, as in the ABCD-BIDS pipeline, the default of 0.08 is used for the “—upper-bpf”), and “–band-stop-min” <min> “–band-stop-max” <max> (set the frequency range used for respiratory motion filtering: PA001: 12–18 breaths per minute (bpm), PA002: 11–17 bpm, PC001&2: 15–25 bpm). Confound regression included 36 nuisance regressors: six motion parameters, mean grayordinate signal, mean white matter signal, mean cerebrospinal fluid signal with their temporal derivatives, and quadratic expansion of six motion parameters, tissue signals and their temporal derivatives. Band-stop filtering is applied to motion parameters prior to the regression. Frequency ranges for the band-stop filter were chosen based on the typical age specific respiration rates suggested in XCP-D’s documentation and, if necessary, adjusted based on individual peaks in respiratory power plots (PA002). Frames with FD >0.3 mm were replaced with interpolated data from surrounding low motion frames before data denoising. For frame censoring for functional connectivity matrices created in subsequent analysis steps, an FD threshold of 0.2 mm was used.

Neonatal images were preprocessed with the infant-abcd-hcp-pipeline (Sturgeon, Snider, et al., 2023), an infant-specific modification of the HCP pipeline (Feczko et al., 2021;Glasser et al., 2013). Segmentations of anatomical images were created using BIBSnet, a deep learning tool specifically trained for infant MRI image segmentation (Hendrickson et al., 2023). These precomputed segmentations were utilized by the infant-abcd-hcp-pipeline during preprocessing. Anatomical images were registered to the MNI infant atlas, and functional data were projected onto the atlas space surfaces. Spin echo fieldmaps were leveraged for susceptibility distortion correction using FSL topup (Andersson et al., 2003). Functional connectivity processing was performed during the DCANBOLDProcessing stage of the infant-abcd-bids-pipeline, which reflects the processing steps performed by XCP-D (interpolation of high-motion volumes, detrending, confound regression using the same 36 nuisance regressors, bandpass filtering). Compared to adults and children, no respiratory filter was applied for the infant data as their respiration rate (30–60 bpm) is higher than half of the Nyquist frequency based on the TR. FD traces were visually inspected as recommended byPower et al. (2012)and it was determined that 0.3 was the noise floor for infants. Runs with less than 30% of remaining data at an FD of 0.3 mm were excluded. After censoring frames with high motion (above FD 0.3 mm) during data preprocessing, PB004 retained 57 min of low motion data (out of 77), PB005 79 min (out of 117), and PB001 112 min (out of 142) with one entire run excluded. From the additional SE data, PB004 retained 72 min (out of 77) and PB005 retained 85 min (out of 123;Supplementary Table 1).

As the infant-abcd-hcp-pipeline is not constructed for ME data, data from all five echoes were optimally combined using Tedana (DuPre et al., 2021;Kundu et al., 2012,^2013^;The tedana Community et al., 2022) before running the pipeline. For the optimal combination, we used the T2* based echo weighting implemented in Tedana, which is also implemented in fMRIprep. In addition, motion regressors were calculated from the first echo and applied to all individual echoes before combining them.

### 2.4 Data analysis

We calculated tSNR, defined as mean intensity over the timeseries divided by the time series standard deviation, in the data with and without NORDIC applied. Averages across runs with >90% low motion data were used for tSNR estimates. This is done to ensure that this metric is not impacted by subjects’ motion while keeping the same amount of frames for each run (no motion censoring is applied to the runs). The amount of averaged runs, therefore, varies by subject and condition (from 2 to 14 runs; seeSupplementary Table 1). Overall data smoothness was estimated from preprocessed BOLD runs using 3dFWHMx (options -combine -detrend -automask -acf) in AFNI (version 16.1.13;Cox, 1996). Examples for smoothed FWHM were created using 3dmerge (-1blur_sigma 1.5 and 2.5). As smoothing can also increase tSNR, to further understand NORDIC as a denoising technique, we additionally investigated tSNR of smoothed SE and SE-NORDIC volumes. Data were smoothed to approximately the same FWHM as ME-NORDIC using 3dBlurToFWHM in AFNI (-FWHM 2.71 for SE and 2.69 for SENORDIC—applied in each direction to achieve similar overall data smoothness in each run). Functional connection strength was computed using a parcel-to-parcel connectivity matrix (parcels for adults and children fromGordon et al. (2016), for neonates fromMyers et al. (2024)).

Reliability was quantified as the grayordinate-by-grayordinate correlation of dense functional connectivity matrices within subject similar toLynch et al. (2020). Dense connectivity matrices were computed without applying any additional spatial smoothing (other than forSupplementary Fig. 18). For constructing reliability curves, data were split in half and consecutive amounts of data compared to the held-out half. To account for natural variations in data quality during one session and across multiple days, the order of runs was randomly permuted 100 times and an average curve calculated. Reliability curves for parcellated data were calculated in the same fashion. The reliability values reported throughout the paper represent the average value of the spatial reliability map for all grayordinates (which includes surface vertices and subcortical voxels) or parcels.

We investigated optimal echo times (T2*) as outputted by Tedana as well as the weights of each echo used for the T2* based optimal echo combination for each grayordinate. As for tSNR estimates, T2* was averaged across runs with >90% low motion data as T2* maps are estimated during data preprocessing before motion censoring.

## 3 Results

### 3.1 ME and NORDIC improved reliability while keeping spatial precision

**Fig. 1.**
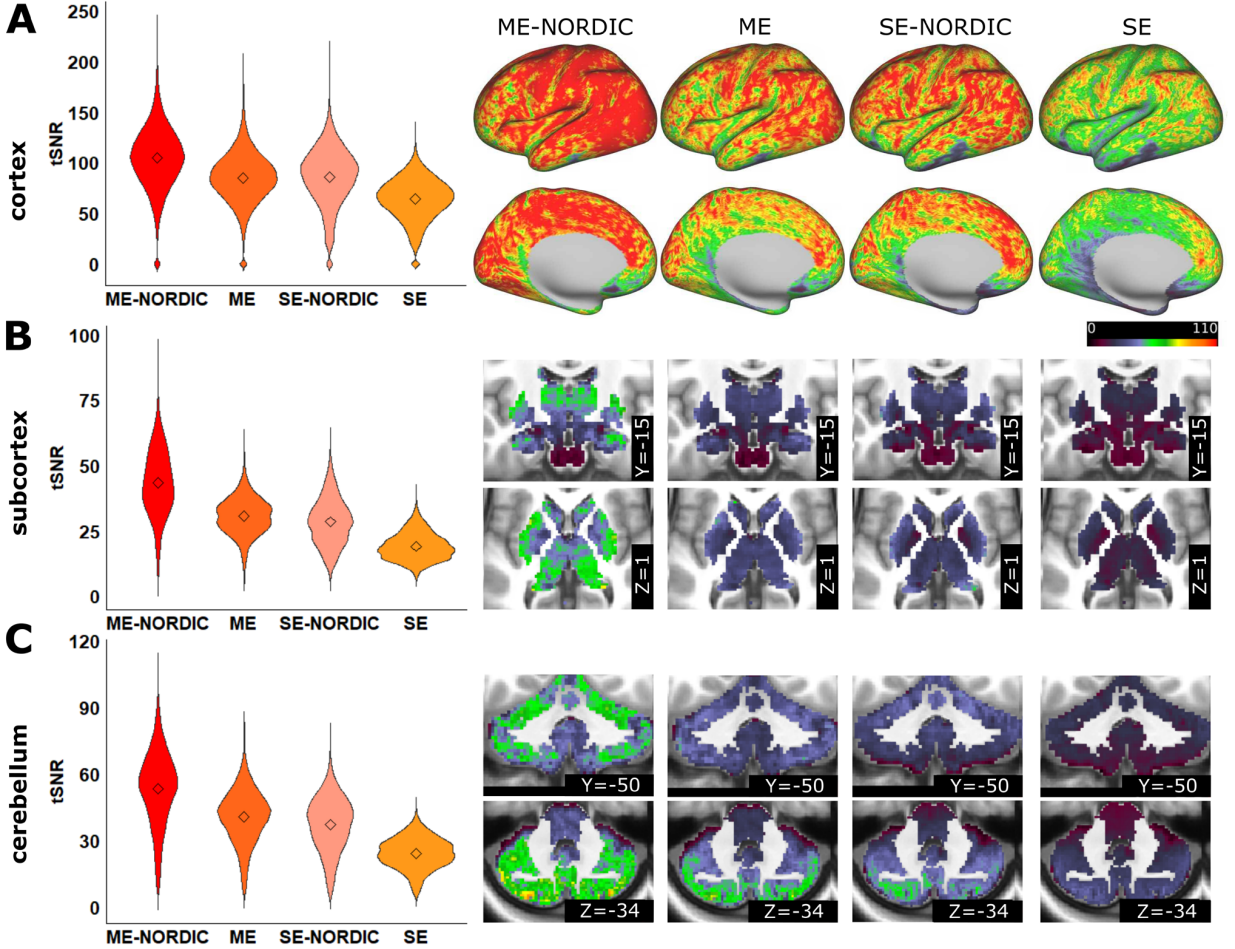
tSNR values for cortical and subcortical structures for SE and ME data with and without NORDIC for adult subject PA001 (average of runs with >90% low motion). (A) Cortex, (B) Subcortex, (C) Cerebellum.

**Fig. 2.**
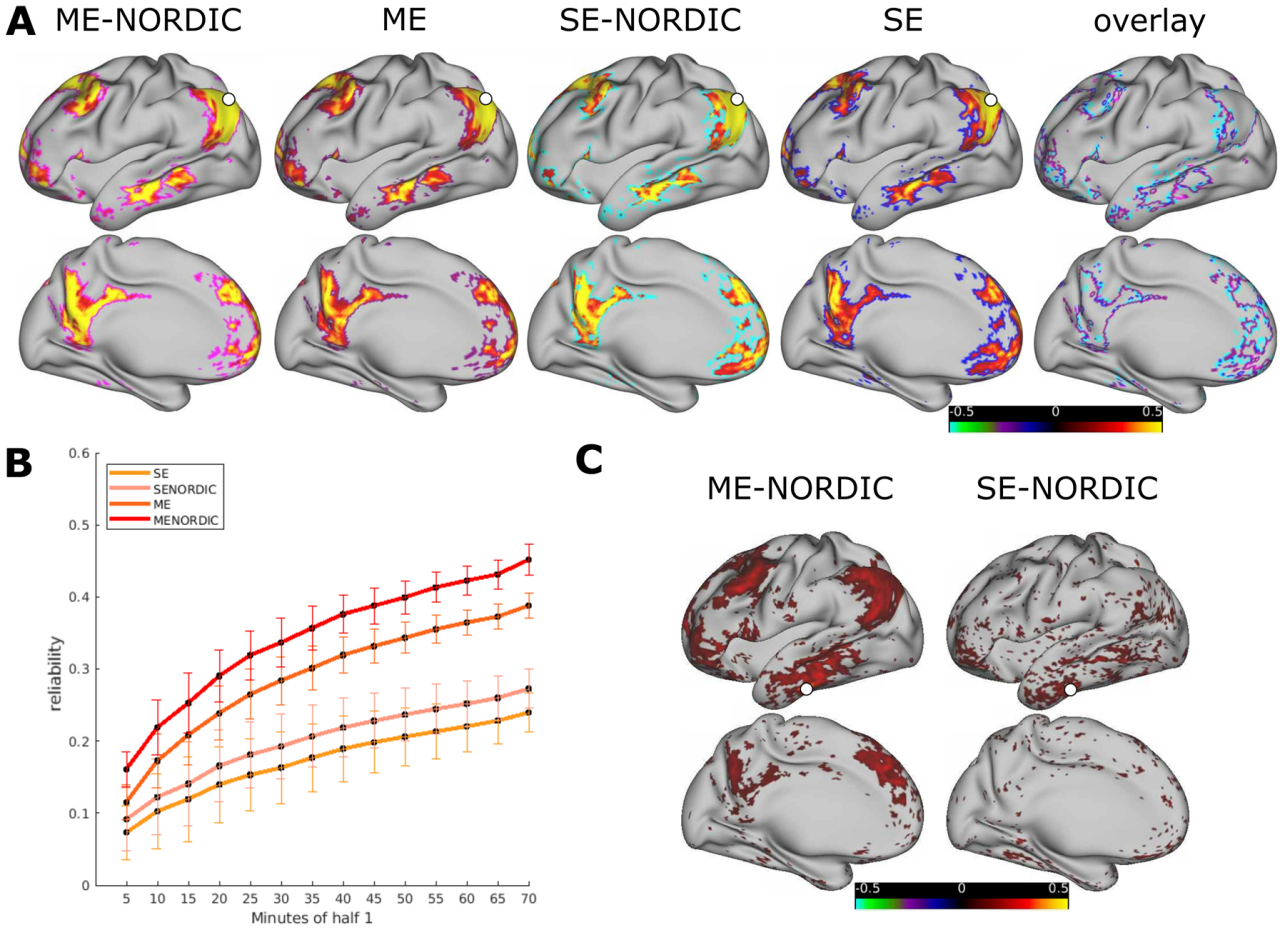
(A) A person’s specific connectivity pattern (adult PA001) remains across SE and ME acquisitions with and without NORDIC. Seed-based connectivity for all four data acquisition conditions (default mode network seed displayed in white). Outlined threshold shows areas of highest connectivity (top 10%, ME-NORDIC outlined in pink, ME in purple, SE-NORDIC in cyan, and SE in blue). Rightmost panel shows the similarity of the spatial extent of high connectivity between conditions. (B) Reliability curves for a split half of the data of PA001. Curves represent the average reliability across all grayordinates and 100 permutations of the run order. Error bars show SD across permutations. (C) ME acquisitions improve signal particularly in areas with high signal dropout which helps to detect connectivity patterns in these areas. Displayed example: seed-based connectivity of inferior temporal seed (seed region displayed in white) comparing ME-NORDIC and SE-NORDIC.

Results from SE and ME data acquisitions in two adult precision imaging participants replicated the recently published (Lynch et al., 2020) increase in tSNR and reliability for ME. Removal of thermal noise with NORDIC furthermore increased tSNR for both SE and ME data. The highest tSNR was obtained with the combination of ME and NORDIC (Fig. 1;Table 1;Supplementary Fig. 1A). Particularly areas with high signal dropout in SE data showed relative gains in tSNR with NORDIC in ME data (Supplementary Fig. 2;Supplementary Table 5). As differences in acquisition sequences between SE and ME impact tSNR, we performed a control analysis, comparing the second echo of the ME sequence (TE2) to the SE sequence (Supplementary Fig. 3). TE2 showed no improvements over SE, highlighting the importance of optimal echo combination in ME. To measure data reliability, the available data for each participant was split in half. The connectivity matrices computed from increasing amounts of consecutive data from one half were correlated to the matrix from the held-out half. Both SE and ME acquisitions had improved reliability with NORDIC while still retaining the person’s specific connectivity structure (Fig. 2;Table 2;Supplementary Fig. 1B). The reliability that can be reached in PA001 with 70 min of SE data (M = 0.24) was reached with 10–15 min of data with ME-NORDIC (Fig. 2B). The advantage of ME was particularly evident when comparing connectivity patterns between SE and ME data in areas with high signal dropout (Fig. 2C). NORDIC increased absolute connectivity strength generally across functional connections (Supplementary Fig. 4;Supplementary Table 3). Importantly, loss in spatial precision for data denoised with NORDIC was minimal (for PA001 FWHM increased from 2.73 to 2.8 for SE-NORDIC and from 3.47 to 3.7 for ME-NORDIC;Supplementary Table 4). Relative to SE data, using ME and NORDIC increased FWHM for PA001 by 0.96, which is minimal relative to commonly used data smoothing approaches (FWHM increased by 3.59 for 1.5 mm of applied spatial smoothing (FWHM = 6.32) and 7.95 for 2.5 mm of applied spatial smoothing (FWHM = 10.68);Supplementary Fig. 5). The impact of the slight difference in smoothness between SE and ME on tSNR for PA001 is portrayed inSupplementary Figure 6. Data smoothing increases tSNR but does not fully account for differences. These results demonstrate an increase in data reliability with minimal loss in spatial precision when combining ME acquisitions with NORDIC.

**Table 1.** tSNR with and without NORDIC for precision imaging participants.

			Non-NORDIC	NORDIC	Percent difference
Adults	PA001	ME	M = 67.93, SD = 31.11	M = 85.11, SD = 37.32	25.29%
		SE	M = 49.23, SD = 26.13	M = 67.52, SD = 34.62	37.15%
	PA002	ME	M = 54.26, SD = 22.09	M = 63.34, SD = 24.83	16.73%
		SE	M = 44.13, SD = 24.25	M = 55.09, SD = 30.11	24.84%
Children	PC001	ME	M = 67.29, SD = 29.69	M = 79.37, SD = 32.55	17.95%
	PC002	ME	M = 72.27, SD = 31.84	M = 84.06, SD = 35.84	16.31%
Infants	PB004	ME	M = 95.4, SD = 38.33	M = 127.66, SD = 48.90	33.82%
		SE	M = 90.2, SD = 38	M = 135.56, SD = 67.63	50.29%
	PB005	ME	M = 68.92, SD = 27.5	M = 81.35, SD = 30.52	18.04%
		SE	M = 68.24, SD = 26.94	M = 83.9, SD = 36.34	22.95%
	PB001	ME	M = 94.21, SD = 39.33	M = 145.84, SD = 53.62	54.8%

SD represents SD across brain grayordinates.

**Table 2.** Split half reliability with and without NORDIC for precision imaging participants.

		Minutes		Non-NORDIC	NORDIC	Percent difference
Adults	PA001	70	ME	M = 0.39, SD = 0.24	M = 0.45, SD = 0.23	16.41%
			SE	M = 0.24, SD = 0.21	M = 0.27, SD = 0.23	13.77%
	PA002	70	ME	M = 0.29, SD = 0.21	M = 0.33, SD = 0.22	14.19%
			SE	M = 0.2, SD = 0.19	M = 0.23, SD = 0.21	15.22%
Children	PC001	80	ME	M = 0.39, SD = 0.26	M = 0.45, SD = 0.27	16.74%
	PC002	70	ME	M = 0.44, SD = 0.23	M = 0.5, SD = 0.23	13.67%
Infants	PB004	35	ME	M = 0.04, SD = 0.05	M = 0.06, SD = 0.07	54.47%
			SE	M = 0.02, SD = 0.03	M = 0.09, SD = 0.09	301.38%
	PB005	25	ME	M = 0.13, SD = 0.13	M = 0.16, SD = 0.15	25.7%
			SE	M = 0.07, SD = 0.08	M = 0.15, SD = 1.15	128.09%
	PB001	50	ME	M = 0.11, SD = 0.12	M = 0.17, SD = 0.15	51.81%

SD represents SD across permutations.

### 3.2 NORDIC improves tSNR and reliability of ME data in developmental samples

ME precision imaging data from two children and three infants were used to assess the translatability of ME-NORDIC benefits to developmental populations, in whom acquisition of large amounts of resting-state data to achieve reliable individual network solutions is less feasible. As in adults, we investigated changes in tSNR, split-half reliability, and strength of functional connections.

**Fig. 3.**
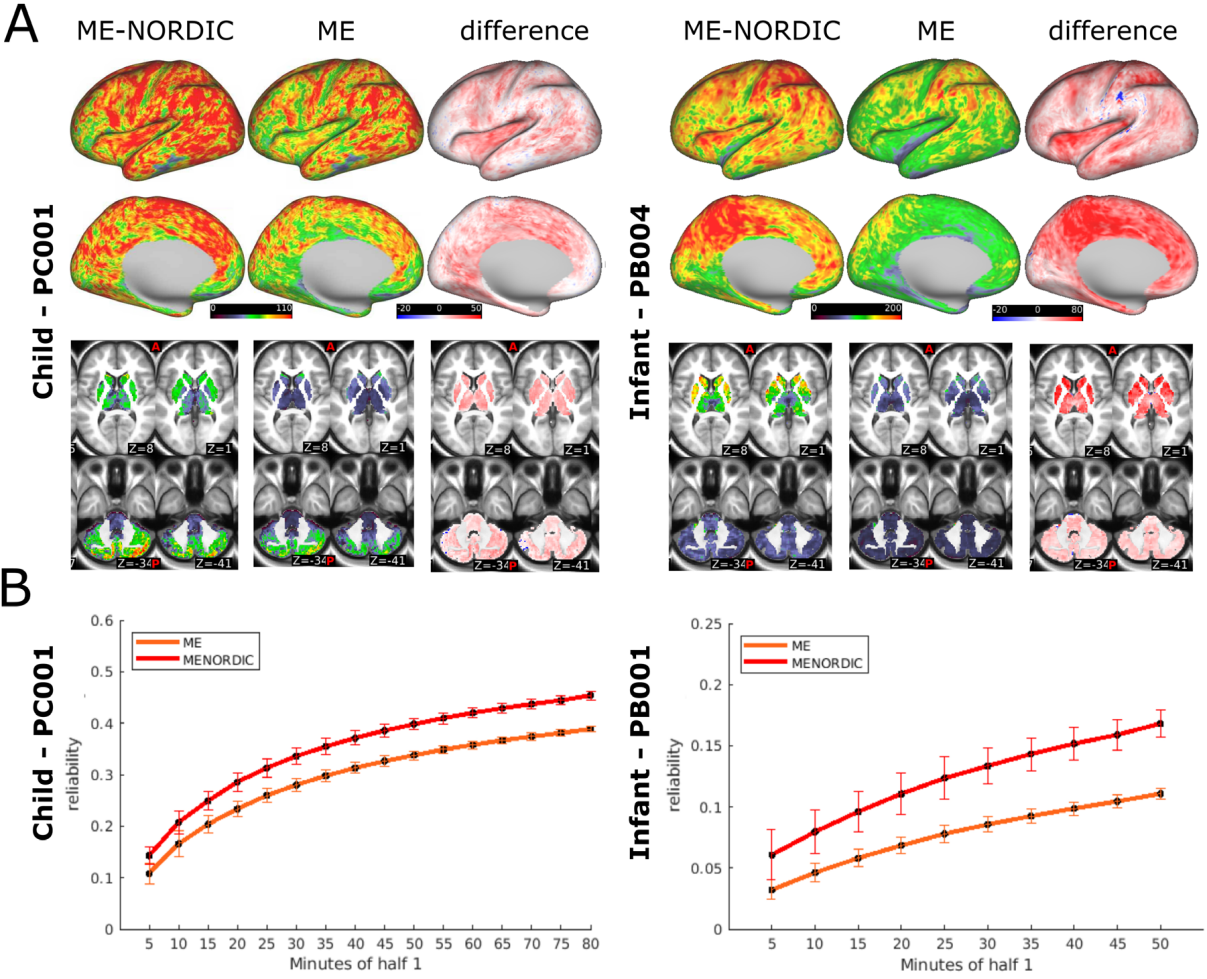
(A) tSNR values for cortical and subcortical structures with and without NORDIC in child participant PC001 and infant participant PB004 (average of runs with >90% low motion). (B) Gains in reliability with NORDIC for PC001 and PB001 (infant with largest amount of low motion ME data). Curves represent the average reliability across all grayordinates and 100 permutations of the run order. Error bars show SD across permutations.

As in adults, we calculated the average tSNR from runs with greater than 90% of low motion data (Supplementary Table 1). We saw an overall improvement in tSNR with NORDIC for all populations (Table 1;Fig. 3A;Supplementary Figs. 7, 8, and 9A). We also observed the spatially specific improvements in tSNR in ME-NORDIC in areas with high signal dropout in SE data in infants (Supplementary Fig. 2;Supplementary Table 5) as well as the lack of tSNR gain for TE2 out of the ME sequence (Supplementary Fig. 10). Similar to adults, split half reliability increased with NORDIC in both children and infants (Fig. 3B;Table 2;Supplementary Figs. 9B and 11).

Furthermore, across all participants and age groups, data denoised with NORDIC showed stronger functional connectivity (Supplementary Table 3;Supplementary Figs. 12 and 13). For this comparison, we looked at parcellated connectivity matrices using the same amount of low motion data for each condition, using an age-specific parcellation scheme for the infants. Similar benefits were also seen in single echo data in infants (Supplementary Figs. 7, 8, 11, and 13).

### 3.3 ME acquisitions in infants provide additional insights regarding brain development

In addition to boosting reliability, ME acquisitions provide insight into fMRI signal properties. The ME data acquisition protocol allowed us to model the T2 decay curve across four/five different time points and estimate T2* relaxation times (Supplementary Fig. 14shows consistency of these estimates with and without the fifth echo). The mean T2* relaxation time across surface vertices for the adults was 48.89 ms for PA002 (SD = 12.23 ms, 5th and 95th percentile = [21.26 ms, 64.75 ms]) and 50.62 ms for PA001 (SD = 12.7 ms, 5th and 95th percentile = [22.23 ms, 65.51 ms]), which is consistent with the adult literature (Fera et al., 2004;Wansapura et al., 1999). In children, the mean T2* relaxation time was 59.34 ms for PC001 (SD = 13.96 ms, 5th and 95th percentile = [27.91 ms, 75.18 ms]) and 58.16 ms for PC002 (SD = 14.08 ms, 5th and 95th percentile = [24.59 ms, 72.64 ms]), showing a slight increase compared to adults.

**Fig. 4.**
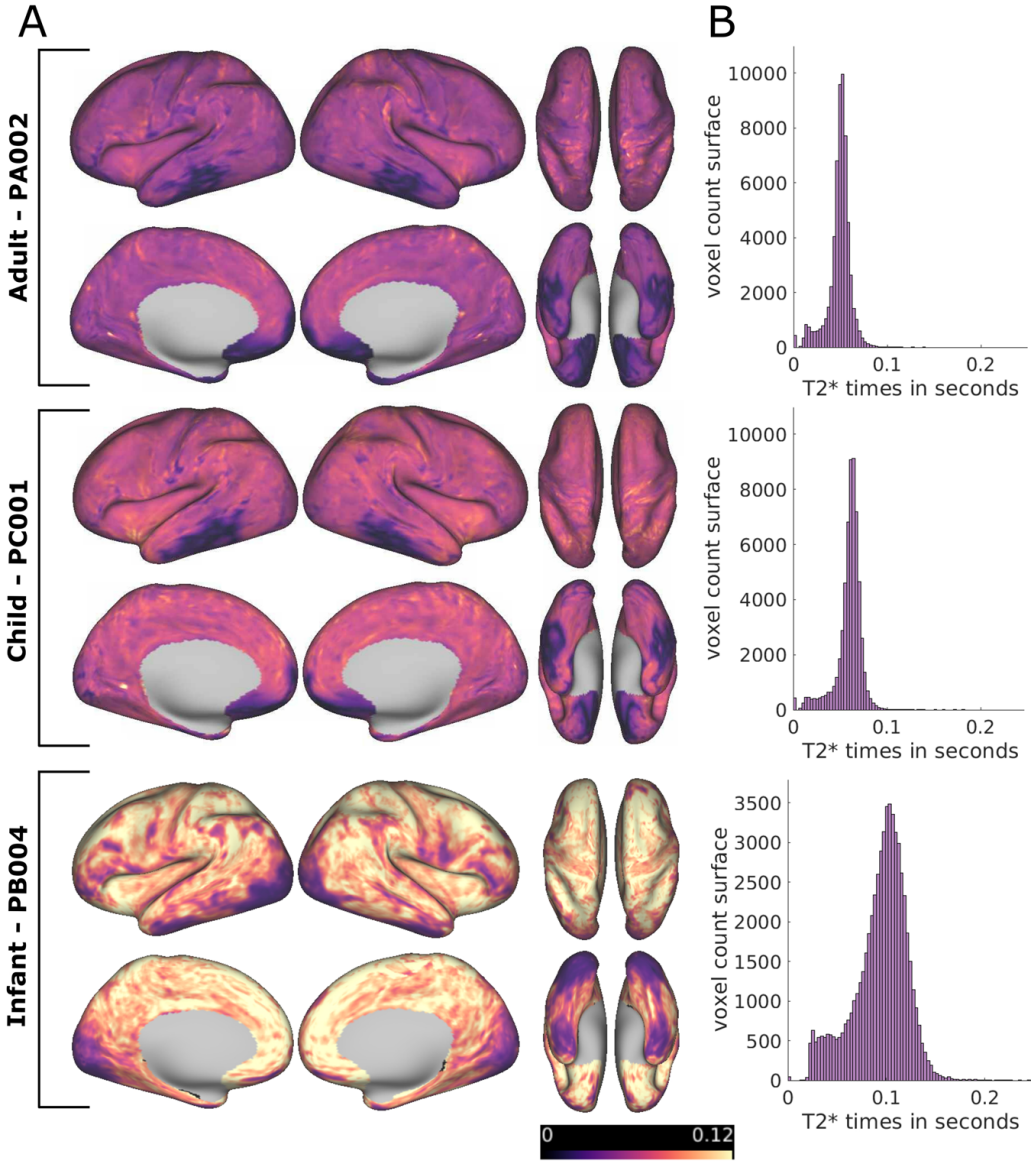
(A) T2* values (in seconds) for PA002, PC001, and PB004 (average of runs with >90% low motion) computed from ME data without NORDIC denoising. (B) Distribution of T2* times from the cortical surface.

T2* relaxation times were longer and more variable in all infants compared to adult or child subjects (Fig. 4;Supplementary Fig. 15). PB004 showed a mean T2* relaxation time of 93.84 ms (SD = 27.04 ms, 5th and 95th percentile = [37.23 ms, 130.22 ms]), PB005 77.49 ms (SD = 30.46, 5th and 95th percentile = [27.43 ms, 124.6 ms]) and PB001 81.51 ms (SD = 27.84, 5th and 95th percentile = [31.91 ms, 124.10 ms]). Application of NORDIC denoising to the data had a minimal and non-systematic impact on the estimate of T2* (Supplementary Fig. 15). The error of the model fit for the T2 decay curves is displayed inSupplementary Figure 16and showed more variability in the infants compared to the adults and children.

### 3.4 Tissue properties in infants affect the optimal echo combination

**Fig. 5.**
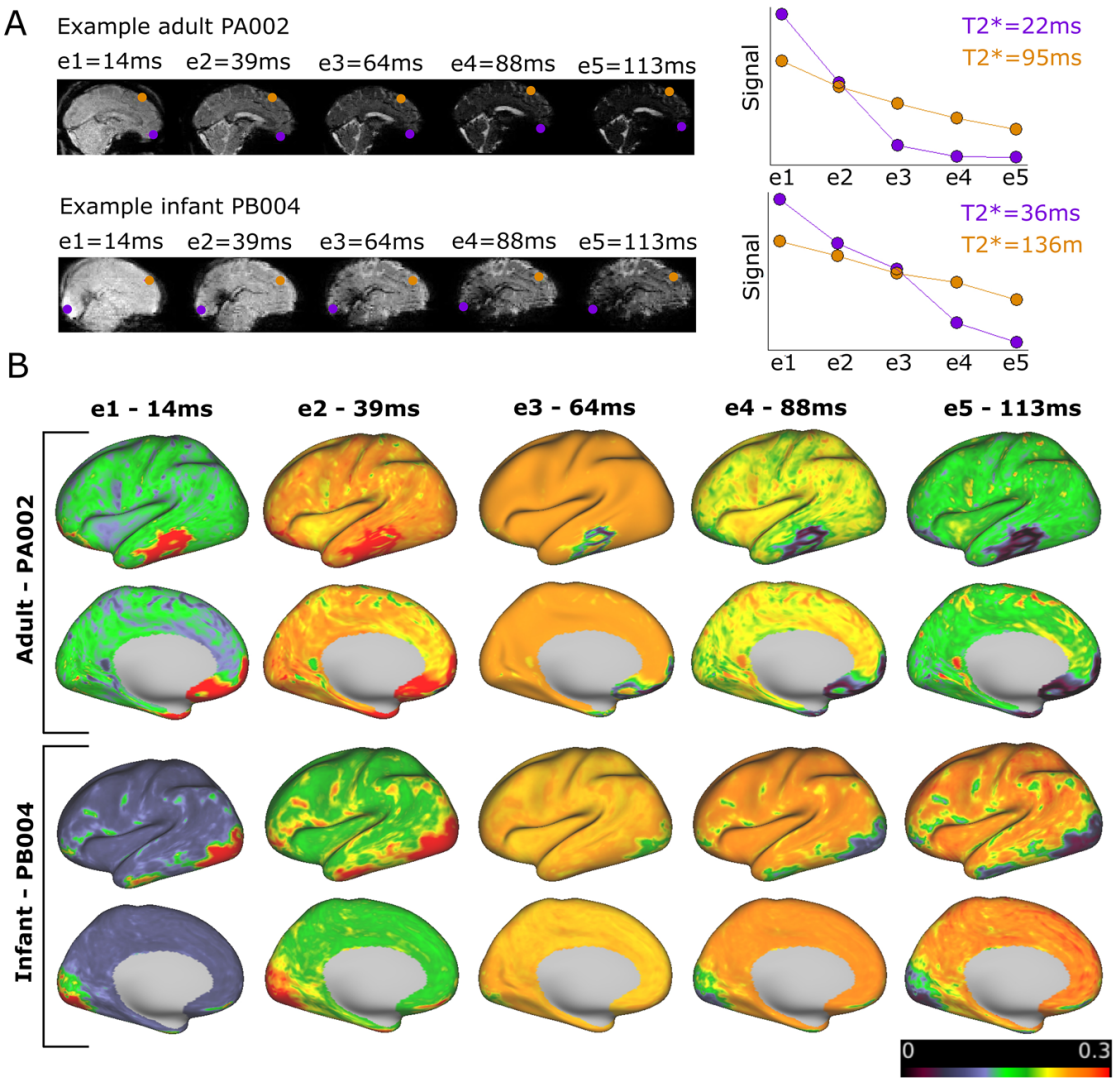
(A) Example for T2 decay curve for PA002 and PB004 at two voxels with short and long T2* over five echoes. (B) Echo weighting distribution across the cortex for all five echoes for an example adult and an example newborn resulting from the T2* maps displayed inFigure 4.

The large difference in T2* relaxation times between infants and adults or children (Fig. 4) highlights an important challenge for methodological advancements for developmental neuroimaging, namely the translatability of findings from adults to brains with fundamentally different properties. In this specific example, the striking difference in T2* relaxation times during early development caused an alteration in echo weighting for the optimally combined ME signal between the different age groups (Fig. 5;Supplementary Fig. 17). Differences in optimally combining ME acquisitions between adult subject PA002 (five-echo sequence) and the infant subjects are summarized inTable 3. The mean normalized weighting per echo in PA002 was highest for the second and third echo while, for the infants, the third to fifth echoes were weighted heaviest. In all subjects, weighting of the first echo was highest in limited areas which is likely related to signal dropout in these areas (Fig. 5). The differences in echo weighting in different age groups affect tSNR, and as a corollary, the impact of NORDIC, as thermal noise contribution relative to measured signal, depends on the echo time.

**Table 3.** T2* times in ms and normalized weights for each echo for the one adult and the three newborns acquired with the five-echo ME protocol.

		T2*	**e1**	**e2**	**e3**	**e4**	**e5**
PA002	mean	48.89	0.17	**0.247**	**0.235**	0.196	0.153
	median	50.71	0.144	0.242	0.243	0.208	0.163
	SD	12.23	0.095	0.026	0.031	0.042	0.043
	5th pct	21.26	0.115	0.215	0.189	0.091	0.041
	95th pct	64.75	0.347	0.31	0.243	0.228	0.2
PB004	mean	93.84	0.101	0.194	**0.232**	**0.240**	**0.233**
	median	98.58	0.085	0.182	0.232	0.251	0.25
	SD	27.04	0.047	0.035	0.008	0.031	0.049
	5th pct	37.23	0.074	0.167	0.224	0.170	0.113
	95th pct	130.22	0.198	0.281	0.242	0.259	0.274
PB005	mean	77.49	0.126	**0.215**	**0.233**	**0.223**	**0.203**
	median	79.73	0.098	0.197	0.234	0.241	0.226
	SD	30.46	0.067	0.046	0.012	0.043	0.064
	5th pct	27.43	0.075	0.170	0.212	0.12	0.062
	95th pct	124.6	0.287	0.319	0.243	0.258	0.271
PB001	mean	81.51	0.114	**0.207**	**0.234**	**0.230**	**0.214**
	median	83.51	0.095	0.194	0.235	0.243	0.232
	SD	27.84	0.054	0.039	0.009	0.035	0.055
	5th pct	31.91	0.075	0.169	0.224	0.146	0.086
	95th pct	124.06	0.239	0.302	0.243	0.258	0.271

Highlighted in bold: echo weightings above 20%.

### 3.5 Competition between reliability and spatial precision

**Fig. 6.**
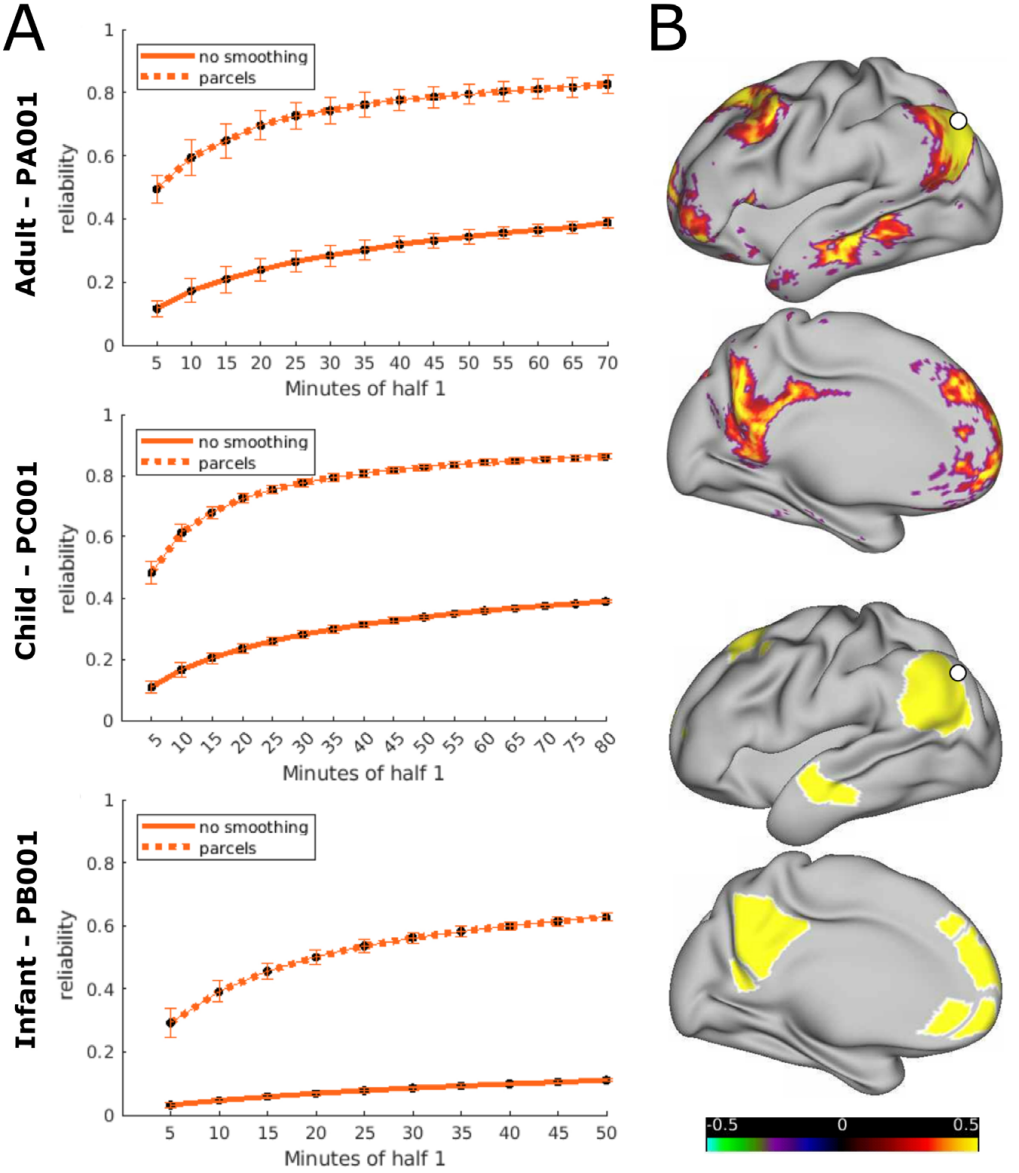
(A) Differences in reliability between dense, unsmoothed, and parcellated ME data without NORDIC (Gordon parcels for PA001 and PC001 and infant-specific parcels for PB001). Curves represent the average reliability across all grayordinates or parcels and permutations. Error bars show SD across permutations. (B) Example for difference in precision between dense unsmoothed and parcellated data (ME data PA001, without NORDIC).

To accurately characterize individual specific networks, one needs to carefully consider the tradeoff between reliability and spatial precision. This tradeoff is illustrated inFigure 6. Reducing spatial precision, for example by parcellating data, fundamentally increases reliability (Fig. 6A, dotted lines vs. filled lines). However, averaging across parcels eliminates the opportunity to uncover precise individual specific patterns (Fig. 6B). Thus, reliability depends on spatial precision. Another example, using 1.5 and 2.5 mm of applied spatial smoothing in data from PA001, is demonstrated inSupplementary Figure 18. This example further demonstrates that reliability curves not only scale with spatial smoothness, as reliability for unsmoothed ME-NORDIC data is higher than for considerably smoothed SE data (see alsoSupplementary Table 4). This highlights the challenge of interpreting absolute reliability values when searching for the ideal amount of scan time. Furthermore, it is important to note that absolute reliability values also depend on the amount of held out data used to calculate them (Supplementary Fig. 19).

## 4 Discussion

In this study, we evaluated potential methodological avenues to improve data quality and reliability to facilitate precision functional imaging, with a specific focus on developmental populations. The two methods evaluated were ME data acquisitions and NORDIC thermal denoising. In adult precision imaging subjects, we replicated prior findings showing improved tSNR and reliability with ME acquisitions (Lynch et al., 2020). Improvements in tSNR with NORDIC denoising have been previously demonstrated for SE fMRI data (Dowdle et al., 2023;Vizioli et al., 2021). Here, we demonstrated the impact of NORDIC on reliability of resting-state functional connectivity and the unique benefit of combining ME and NORDIC for precision functional imaging. Expanding these findings to children and infants, we replicated the benefits of NORDIC on ME data. We furthermore used the unique feature of ME acquisitions to evaluate T2* relaxation times in all included age groups. T2* values calculated from ME data were longer and more variable in infants compared to adults. This led to a differential optimal echo combination, with heavier weighting of later echoes for the infant datasets. The ability of ME data to capture data from TEs that are closer to the infant’s T2* could be of great advantage for infant fMRI. Yet, the impact of this differential echo weighting on optimally combined ME data needs further investigation. Lastly, we demonstrated some caveats of measuring data reliability, namely its dependence on spatial precision and the available held-out data.

### 4.1 PFM is feasible across development with developmentally-specific consideration

The datasets used to investigate the impact of methodological advances in the present study show that extended fMRI data acquisitions are feasible in children and even in newborn infants. For infants, collecting data over consecutive days to minimize brain developmental changes within the data acquisition period is possible. This mitigates one major constraint for precision imaging in infants, that is, that the brain develops rapidly in early infancy (e.g.,Kostović et al., 2019). Thus, sessions for data collection need to be spaced as closely as possible in order to concatenate data without introducing variability caused by developmental changes in the brain. Even though the overall process itself was feasible, we found that motion, for example during active sleep phases or induced by spontaneous waking, is a greater challenge for this age group, than for adults or 10 year-old children. Further, motion can vary substantially between individual infants, as can be seen by the difference between subjects in the amount of retained data (PB004: ME: 82%, SE, 95%; PB005: ME: 67%, SE: 67%; PB001: 83%;Supplementary Table 1).

### 4.2 NORDIC thermal noise reduction improves data quality

Our results showed an overall benefit in data quality when using NORDIC denoising in the adult, child, and infant brains. We saw an increase in tSNR as well as in the strength of functional connections and split half reliability with NORDIC. Compared to using spatial smoothing as a means to remove noise in the data and increase reliability, the application of NORDIC is preferable in order to retain spatial precision in the data (Dowdle et al., 2023;Vizioli et al., 2021). This is particularly important for infants because of their smaller brain size.

The present results suggest that NORDIC could be a valuable addition to future studies, especially since it does not come at a significant cost (other than increased data storage), as it only additionally requires saving the phase data (along with the standard magnitude images). Both the acquisition of additional noise frames at the end of the scan and the usage of the default parameters (as used for PB001) proved to be beneficial. The default parameters are generally more conservative (i.e., resulting in the removal of less principal components) than the estimated threshold when using the noise frames, which allow for a 5–10% higher threshold based on a more direct estimate of thermal noise. While little effect of using the threshold based on the additional noise frames has been found in 7T auditory fMRI (Faes et al., 2024), 3T imaging might benefit from these higher thresholds (Knudsen et al., 2023).

### 4.3 Advantages of ME acquisitions

ME fMRI showed improvements in data quality and reliability compared to SE acquisitions, in all subjects and across all age groups.

The evaluation of ME data acquisitions in newborn infants confirmed the findings of previous studies that showed overall longer T2* relaxation times in newborns compared to adults (Rivkin et al., 2004;Williams et al., 2005). It should be noted that T2* times vary with magnetic field strength (Peters et al., 2007) and many past infant studies used a 1.5T magnet (e.g.,Leppert et al., 2009;Rivkin et al., 2004). In addition to overall longer T2* relaxation times, our evaluation showed that newborn brains exhibit greater spatial variability of T2* compared to adults, which may be in part due to the combination of longer T2* times in developing tissues and short T2* times in areas impacted by high magnetic susceptibility effects (e.g., air tissue interfaces). This result highlights the challenge of finding an optimal echo time for data acquisition within a given developmental age group (e.g., newborns) and supports the idea that ME fMRI could be a useful tool for imaging during brain development. Developmental studies (particularly longitudinal studies) could particularly benefit from using ME as it allows to use T2* based optimal echo weighting to dynamically adapt to developmental changes. Furthermore, the ability to estimate T2* from ME acquisitions opens up the possibility to measure other tissue properties that may be especially salient to developmental changes, for example, brain iron, which can be quantified by 1/T2* (R2*; e.g.,Larsen et al., 2020).

However, the impact of increased weighting of data from later echoes, which, as a function of the T2 decay curve, have weaker signals, needs to be carefully investigated. Even though BOLD activation can be detected across a wide range of TEs, results vary in sensitivity (Posse et al., 1999). Further investigations using a task fMRI design could help to better understand the use of ME acquisitions in infants. For example,Goksan et al. (2017)acquired data in infants experiencing noxious stimuli at the heel with five different SE protocols in order to identify the echo time with the maximal task contrast. Using a task design with an ME acquisition in infants could help to determine the range of echo times that optimally characterize task activation and which echoes potentially just introduce more noise. Our results, which show a large variability in T2* relaxation times in the newborn brain, suggest that it is unlikely that there is a single optimal echo time for infant fMRI. In any case, the number and timing of echoes in an ME fMRI protocol for developmental studies can still be further optimized. It will be important to consider the balance between the number of echoes, the spatial and temporal resolutions, and the acceleration factors, which are all interdependent with respect to the overall functional contrast to noise ratio. However, across all populations, our findings demonstrate the usefulness of including a relatively short TE to capture signals in areas with high signal dropout (Fig. 2C).

### 4.4 Advancing PFM by combining ME and NORDIC

Findings from the present study highlight the unique advantage of combining ME and NORDIC for PFM, maximally increasing reliability without sacrificing spatial precision, exactly fitting to the needs of PFM, and potentially leading to improvements in other fMRI study designs as well. The large improvements of tSNR in subcortical regions are particularly promising for studies of systems that span subcortical to cortical areas and their functional development. Relative gains in tSNR and reliability with NORDIC, however, vary between individuals and between SE and ME data. Our comparisons suggest that NORDIC does not show the exact same additive effect in SE and ME data, as the optimal combination of data from multiple echoes also removes a fraction of thermal noise (compare gains inTable 1). At the same time, by recovering signals in regions with high signal dropout (seeSupplementary Figs. 1, 7, and 8), ME acquisitions allow for larger gains with NORDIC in these particular areas (Supplementary Fig. 2;Supplementary Table 5), which could be seen as a multiplicative or interactive effect. In this context, other factors additionally need to be considered like the noise introduced by motion. The potential increase in tSNR by removal of thermal noise is limited for data in which tSNR is already compromised by motion. The infant data provide the most compelling evidence for this dependency. For example, PB004 SE and PB001 had multiple runs close to 100% low motion and showed the largest gains with NORDIC compared to the other infants that had more data compromised by motion (e.g., PB005).

Another factor that may affect the impact of NORDIC on tSNR is related to subject differences in the weighting of each echo during optimal combination of ME data. NORDIC is applied to data from each echo separately before optimally combining the data. Due to the signal decay associated with longer TEs, the relative contribution of thermal noise is different for each echo. Thus, NORDIC may have a variable benefit on tSNR depending on the relative contribution of different TEs to the optimally-combined data. Further investigations into the interaction of NORDIC and TE and the impact on optimally combined data need to be done to fully understand the combination of ME and NORDIC. This might be particularly impactful for studying infants with ME acquisitions due to their differences in echo weighing compared to children or adults.

### 4.5 Considerations for investigating tSNR

In this study, tSNR was used as one metric for evaluating the quality of data obtained using different sequences. However, it is important to acknowledge the complexities and limitations inherent in interpreting tSNR. tSNR is defined voxel-wise over time as the ratio between the mean signal and the temporal standard deviation. The temporal standard deviation represents the sum of contributions from true BOLD signal, thermal noise, and physiological noise (i.e., head motion, respiration, pC02 fluctuations). tSNR will vary with data acquisition parameters such as spatial resolution, echo time, acceleration factors (e.g., multi-band, IPAT), flip angle, and field strength (Triantafyllou et al., 2005). Thus, the interpretation of tSNR comparisons between SE and ME data presented in this study should be considered in light of differences in sequence acquisition parameters, which cannot all be held constant during optimization (seeSupplementary Table 2). The ME and SE sequences were matched in spatial resolution (2 mm). However, variables such as TR, flip angle, and MB factor were not matched between all sequences.

To account for one possible parameter affecting signal quality, that is, TR (hence, flip angle), an additional analysis (Supplementary Figs. 3 and 10) compared tSNR of the second echo of the ME data to the SE data (TE2 = 39 ms and TE-SE = 37 ms). These two time series showed comparable tSNR, whereas the tSNR obtained with all echoes in the ME data was higher. This result suggests that the flip angle or slower TR of the ME sequence was not the major determinant of tSNR improvement. On the other hand, it should be noted that tSNR does not capture the potential benefit of a shorter TR in the context of signal denoising. Specifically, BOLD signals of neural origin, which are restricted to low frequencies (<0.2 Hz;Anderson, 2008), may be more easily separated from physiologic artifacts (e.g., head motion, respiration;Fair et al., 2020) with faster TRs. Signal improvements from denoising of this type will not necessarily be reflected in tSNR, but may be reflected in analysis of functional connectivity reliability (one of our other evaluation metrics).

tSNR is also strongly dependent on spatial smoothing (Triantafyllou et al., 2006). Thermal noise is spatially uncorrelated, whereas physiological noise is partially spatially correlated. Therefore, spatial smoothing improves tSNR to the extent to which tSNR is limited by thermal noise. Similarly, NORDIC improves tSNR by reducing unstructured, that is, thermal, noise. In effect, NORDIC enables high spatial resolution fMRI with less postprocessing spatial smoothing (seeSupplementary Fig. 5for illustration of how much smoothing decreases spatial precision relative to NORDIC). Notably, another analysis comparing ME to SE data (NORDIC and non-NORDIC), controlling for spatial smoothness, demonstrated that NORDIC had substantially increased tSNR in comparison to non-NORDIC data with similar smoothness (Supplementary Fig. 6, bars 1 and 2 versus bars 3 and 4). In contrast, in this analysis, tSNR was very similar in ME data in comparison to SE data (either NORDIC or non-NORDIC) after equating smoothness (bars 1 versus 2 and 3 versus 4). Thus, optimally averaging data over multiple echoes may improve overall tSNR only slightly in excess of spatial smoothing. As discussed in the previous section, there are some spatially specific effects though.

### 4.6 Considerations for investigating reliability

The quantification of reliability in this study followed the example of (Lynch et al., 2020) who performed vertex/voxel-wise correlations between dense connectivity matrices. It should be noted that reliability curves based on the correlation of entire vectorized parcellated matrices as presented in previous literature (Gordon et al., 2017;Laumann et al., 2015) yield higher values overall, which is also reflected in our parcel-wise example inFigure 6A.

The lack of a plateau in the curves we constructed indicates that the available data were not sufficient to act as suitable “ground truth” held out data. This is a major difference between our analysis choice of using split-halfs andLynch et al. (2020), who used the correlation of the connectivity matrix of one run to the one from all other available runs of one subject (up to 5.5 h of held-out data) to evaluate reliability. More generally speaking, these limitations highlight that reliability measures depend on data quantity and spatial precision, likely among other factors (e.g., TR). Interpretation of relative reliability must consider these factors when comparing values between different acquisition and analysis conditions (seeFig. 6;Supplementary Figs. 18 and 19).

However, even considering the limitations to reliability quantification discussed above, reliability curves in infants seem to be overall lower in magnitude compared to children or adults. This is in line with findings byMoore et al. (2023)andSylvester et al. (2022)who suggested that larger amounts of data are needed for precision imaging in infants compared to adolescents or adults. The need for longer acquisition times in infants may be explained by the general factors challenging infant imaging, such as increased motion, smaller head size relative to voxel size, increased distance to the receiver coils, or the lack of fully optimized infant-specific processing pipelines (Dubois et al., 2021;Korom et al., 2021). It may, however, be, in part, related to more biological factors, including the variability in behavioral states in newborns during scanning (quiet sleep, active sleep, wakefulness) compared to adults who are usually awake, fixating at a crosshair. Another source of variance could be small, but real, brain developmental changes across the recording days. Given the reliability limitations in infant precision functional imaging, working at the level of infant-specific parcels (Myers et al., 2024;Wang et al., 2023) or individual networks (Moore et al., 2023) could help to improve reliability at the expense of spatial precision. Another approach would be to use alternative methods to increase overall SNR, such as higher magnetic fields (Annink et al., 2020), which could mitigate partial volume effects by allowing for smaller voxel sizes more fitting to the smaller infant brains, or infant-specific coils (Hughes et al., 2017;Keil et al., 2011;Lopez Rios et al., 2018).

### 4.7 Limitations and outlook

One major advantage of ME fMRI is the possibility of using MEICA for T2* based denoising (Kundu et al., 2012,^2013^;Lynch et al., 2020). This is a feature we were not able to use in the present study, as thresholds commonly used for MEICA are not applicable in our infant data. This is likely due to the developmental differences in T2* and will need further investigation. Given the promise of ME acquisitions for developmental neuroimaging, the development of an infant-specific MEICA version could be valuable for the field, accompanied by further investigations into the effect of MEICA on spatial precision.

Furthermore, the present investigation and findings are limited as they only explored one ME fMRI protocol. More thorough investigations using different ME protocols particularly within infant subjects, and potentially even within longitudinal studies, will exploit the advantages and disadvantages of a given combination of echoes. A longitudinal approach could characterize the precise evolution of T2* relaxation times, informing the optimization of age-specific protocols. The ME protocol used in this study uses a high acceleration factor (MB = 6 with IPAT = 2) which represents a risk of introducing spurious spatial correlations due to “slice leakage”. To prevent this, we used split-slice grappa reconstruction for signal unaliasing (vs. slice-grappa) which was shown to almost entirely suppress false positive activations due to slice leakage even for accelerations this high (Todd et al., 2016). Yet, artifacts are still likely to occur during more severe signal changes like during eye or head movements (McNabb et al., 2020).

Moreover, in the present study, we did not elaborate on the role of well-trained MR operators in enhancing reliability by optimal positioning of participants in the coil and minimizing motion during data acquisition. Development of comprehensive training materials and guidelines will mitigate some of the challenges even before the availability of adequate data processing and analysis methods. As mentioned, the limitations of the present work highlight the need for more developmental specific methods developments, in particular on the avenue toward Precision Functional Mapping during early development.

## 5 Conclusions

The present work demonstrates the utility of combining ME acquisitions with NORDIC denoising for PFM. Translating these methodological advances to developmental populations, ME data acquisitions show high promise for infant imaging, which needs further investigation. There are still gaps in our understanding of the best techniques for developmental brain imaging, motivating the development of further age-specific methodological advances as we work toward broadly applicable and robust precision functional imaging across the lifespan.

## Supplementary Material

Supplementary Material

## Data Availability

Data can be made available upon request, given a formal data-sharing agreement is set up by the institutions involved. Code is available from the following repository:https://github.com/DCAN-Labs/code_infant_me_nordic_paper.
