# Supplementary material for: Multi-echo acquisition and thermal denoising advances precision functional imaging
Source: Imaging Neurosci (Camb). 2025 Jan 9;3:imag_a_00426. doi: 10.1162/imag_a_00426 (PMC12319974; doi:10.1162/imag_a_00426)
Supplement: Supplementary Material [file imag_a_00426-supp.pdf]

Supplementary Material

Multi-echo Acquisition and Thermal Denoising Advances Precision Functional Imaging

Julia Moser[1], Steven M. Nelson[1,2], Sanju Koirala[1,3], Thomas J. Madison[1], Alyssa K. Labonte[4], Cristian Morales Carrasco[1], Eric Feczko[1], Lucille A. Moore[1], Jacob T. Lundquist[1], Kimberly B. Weldon[1], Gracie Grimsrud[1], Kristina Hufnagle[1],Weli Ahmed[1], Michael J. Myers[4], Babatunde Adeyemo[5], Abraham Z. Snyder[5,6], Evan M. Gordon[6], Nico U. F. Dosenbach [5,6,7,8,9], Brenden Tervo-Clemmens[1], Bart Larsen[1,2], Steen Moeller[10], Essa Yacoub[10], Luca Vizioli[10], Kamil Uğurbil[10], Timothy O. Laumann[5,#], Chad M. Sylvester[4,6,11,#], Damien A. Fair[1,2,3,#]

[1] Masonic Institute for the Developing Brain, University of Minnesota, Minneapolis, MN, USA

[2]Department of Pediatrics, University of Minnesota, Minneapolis, MN, USA.

[3] Institute of Child Development, University of Minnesota, Minneapolis, MN, USA

[4] Department of Psychiatry, Washington University in St. Louis, St. Louis, MO, USA

[5] Department of Neurology, Washington University School of Medicine, St Louis, MO, USA

[6] Department of Radiology, Washington University in St. Louis, St. Louis, MO, USA

[7] Department of Psychological and Brain Sciences, Washington University in St. Louis, St Louis, MO, USA.

[8] Department of Pediatrics, Washington University School of Medicine, St Louis, MO, USA

[9] Department of Biomedical Engineering, Washington University in St. Louis, St Louis, MO, USA

[10] Center for Magnetic Resonance Research (CMRR), University of Minnesota, Minneapolis, MN, USA

[11] Taylor Family Institute for Innovative Research, Washington University in St. Louis, St. Louis, MO, USA

#These authors have contributed equally to this work

Supplemental methods

For anatomical references, a T1 weighted scan (PA001 & children: TR = 2.5s, TE = 2.9ms, resolution = 1 x 1 x 1 mm, flip angle = 8°, infants: TR = 2.4s, TE = 2.2ms, resolution = 0.8 x 0.8 x 0.8 mm, flip angle = 8°); and a T2 weighted scan (PA001 & children: TR = 3.2s, TE = 565ms, resolution = 1 x 1 x 1 mm, flip angle = 120°, infant: TR = 4.5s, TE = 563ms, resolution = 0.8 x 0.8 x 0.8 mm, flip angle = 120°); was acquired for all participants. PA002's anatomical references were taken from a previously acquired (now publicly available) dataset (T1w: TR = 2.5s, TE = 1.81ms, resolution = 0.8 x 0.8 x 0.8 mm, flip angle = 8°; T2w: TR = 3.2s, TE = 564ms, resolution = 0.8 x 0.8 x 0.8 mm, flip angle = 120°) (Gordon et al., 2017).

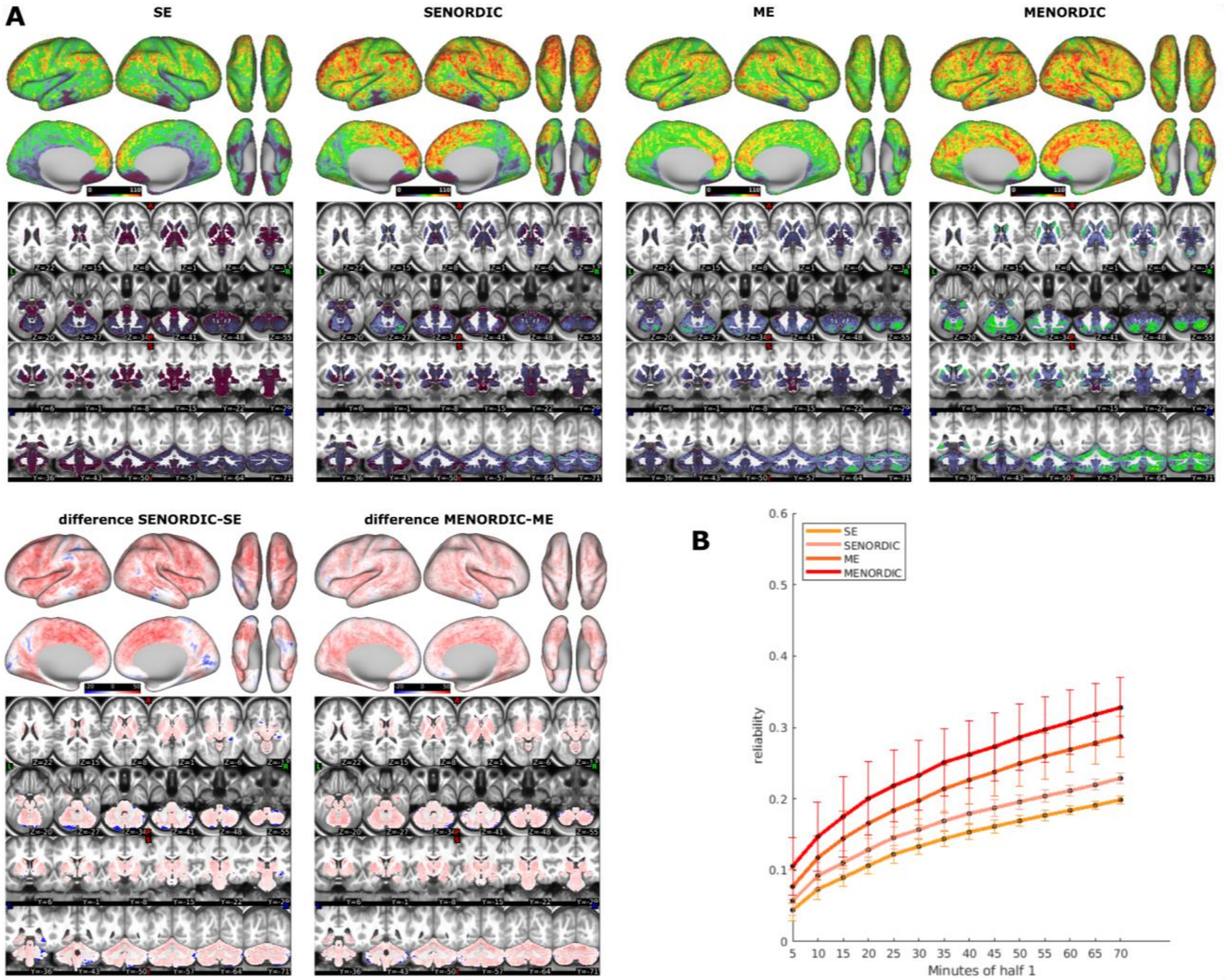

Figure S1: A) tSNR values for cortical and subcortical structures for SE and ME data with and without NORDIC for PA002 (average of runs with >90% low motion). B) Reliability curves for a split half of the data of PA002. Curves represent the average reliability across all grayordinates and 100 permutations of the run order. Error bars show SD across permutations.

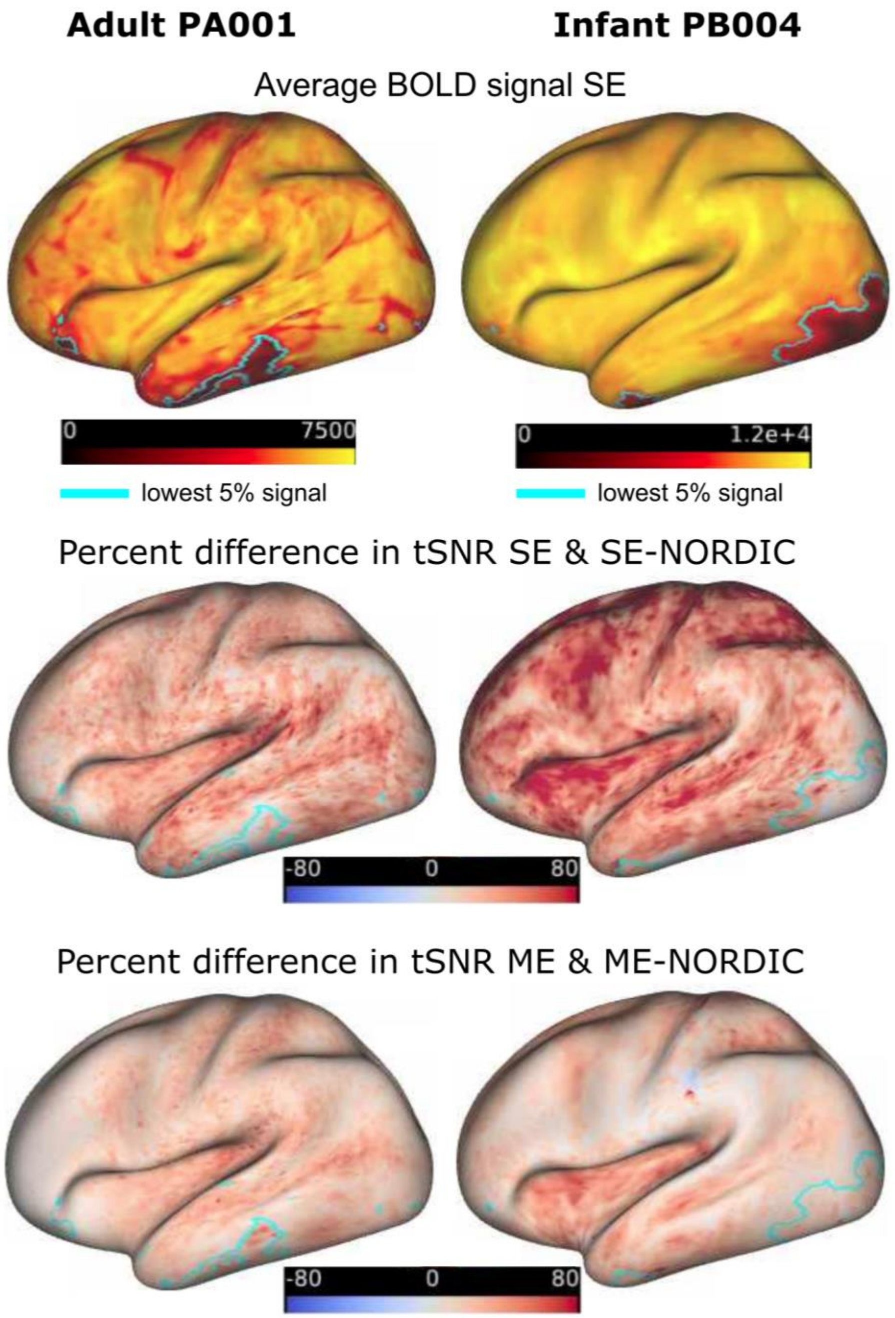

Figure S2: Example of spatial specificity of improvements of tSNR with NORDIC between SE and ME (PA001 & PB004). The mask is defined as regions with lowest 5 % of signal, representing regions with high signal dropout in SE data. Signal in these regions is recovered in ME data (Figure 2C; Lynch et al., 2020). tSNR differences for all SE and ME participants are summarized in Supplementary Table 5.

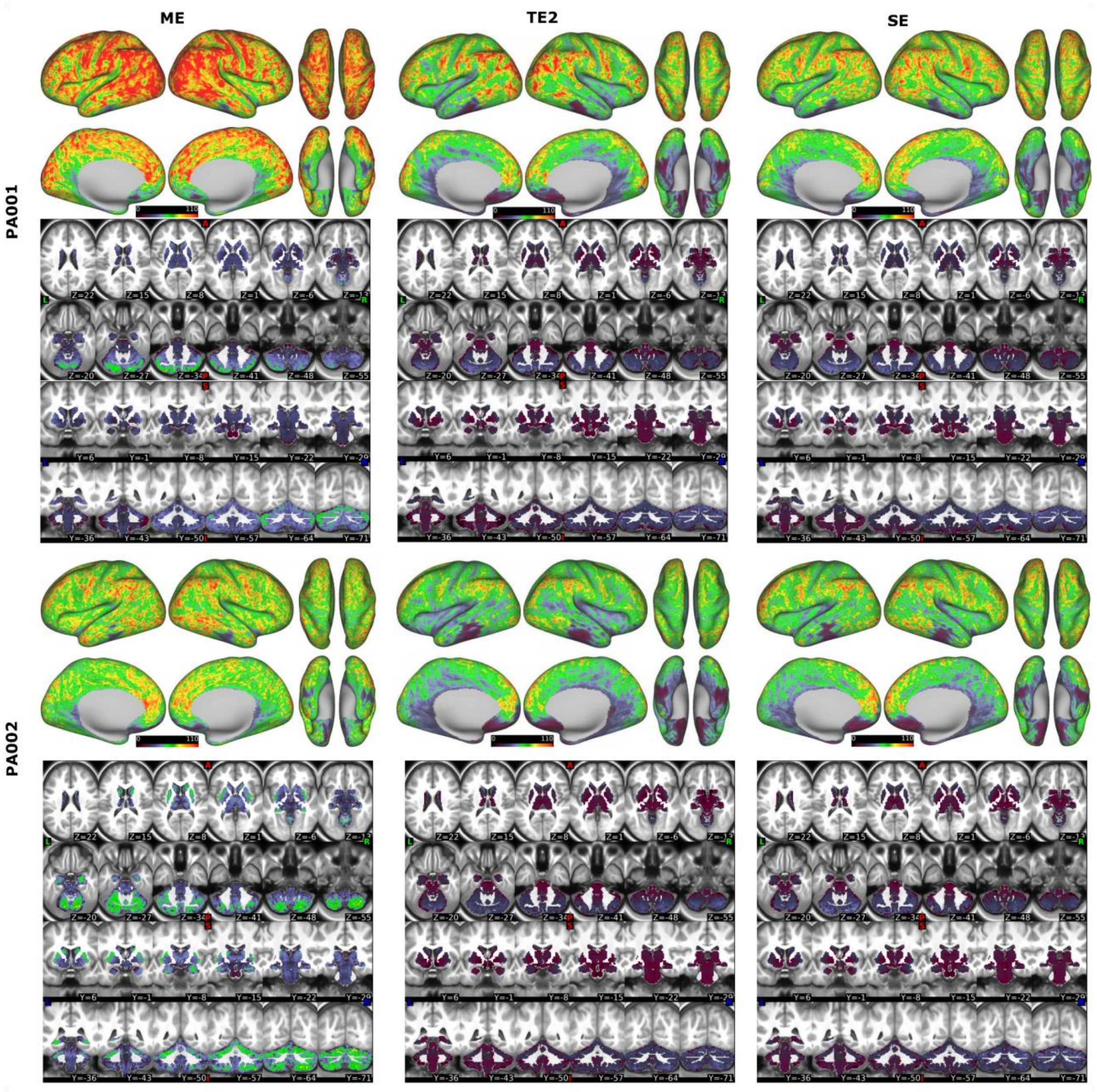

Figure S3: tSNR comparison between ME, SE and TE2 (second echo of ME data only). This comparison addresses whether the differences in TR or flip-angle between ME and SE account for some of the improvements in tSNR seen for ME. PA001 tSNR for ME is highest (M = 67.93, SD = 31.11) compared to TE2 (M = 47.55. SD = 27.26) or SE (M = 49.23, SD = 26.13). PA002 tSNR for ME is highest (M = 54.26 , SD = 22.09) compared to TE2 (M = 40.78, SD = 22.39) or SE (M = 44.13, SD = 24.25). TE2 shows no improvements over SE.

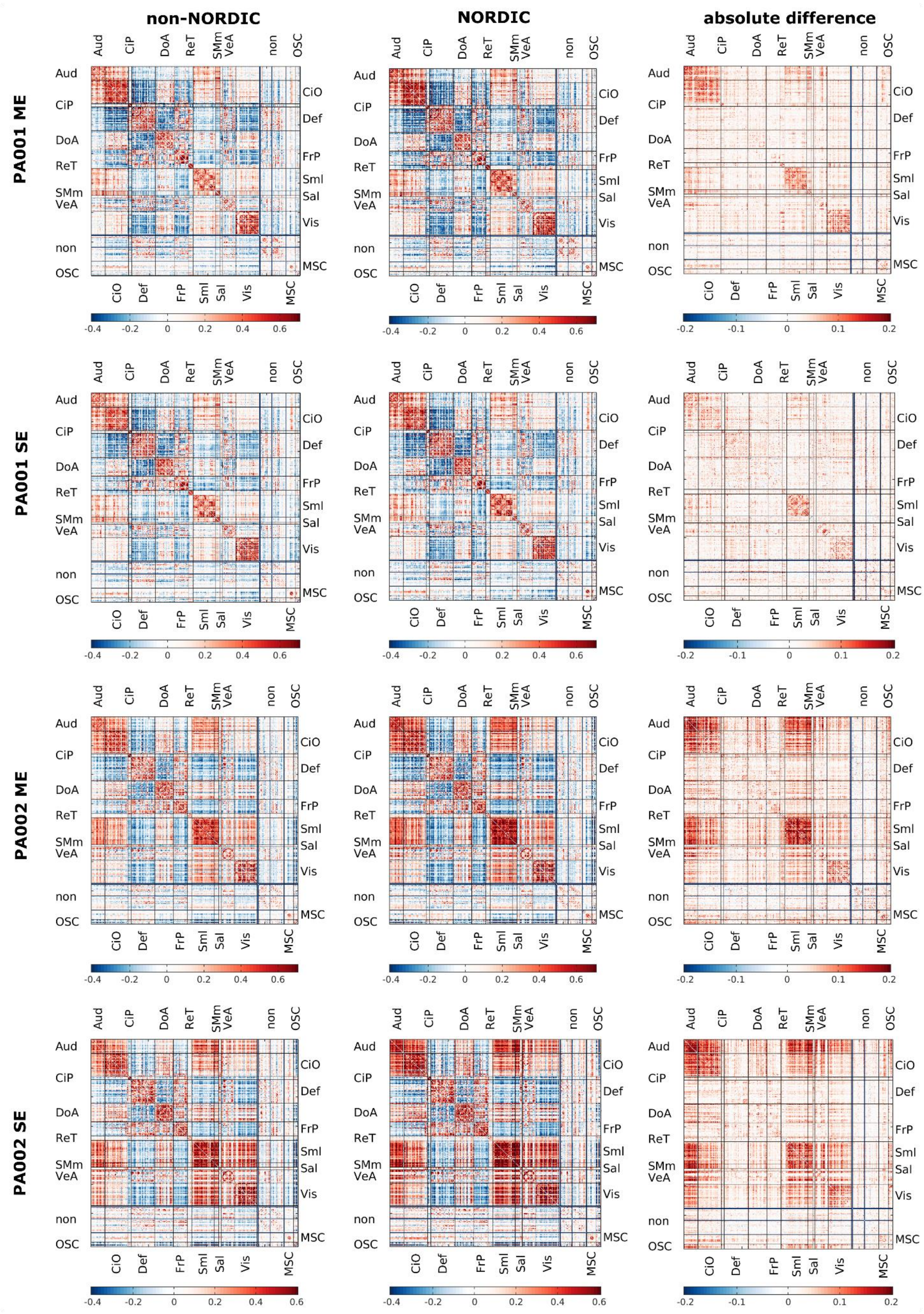

Figure S4: Connectivity matrices from parcellated time series (Gordon parcels) showing the increase of connectivity strength with NORDIC for both ME and SE data in adults.

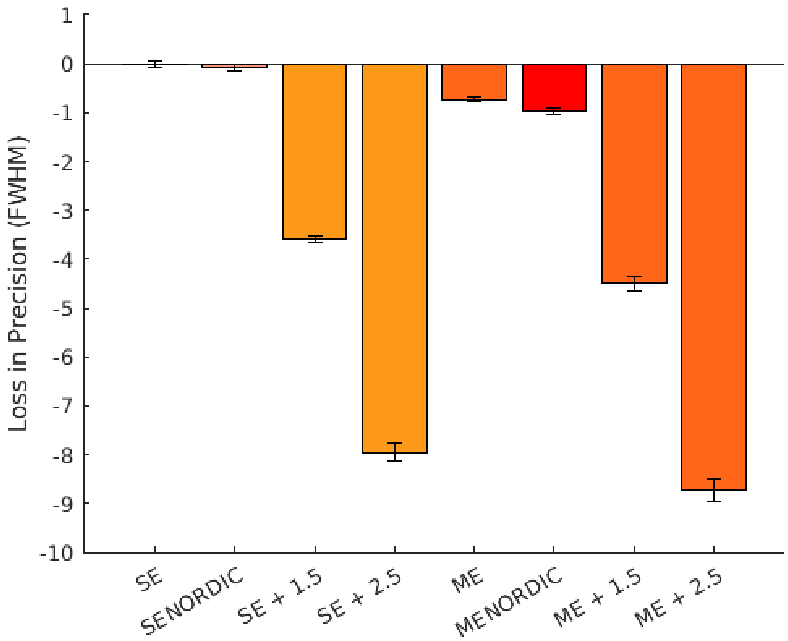

Figure S5: loss in spatial precision with ME and NORDIC compared to commonly used smoothing kernels (σ = 1.5mm and σ =2.5mm) in example subject PA001. Spatial smoothness quantified by full width half max (FWHM) of SE data is used as reference. Error bars represent standard deviation across runs.

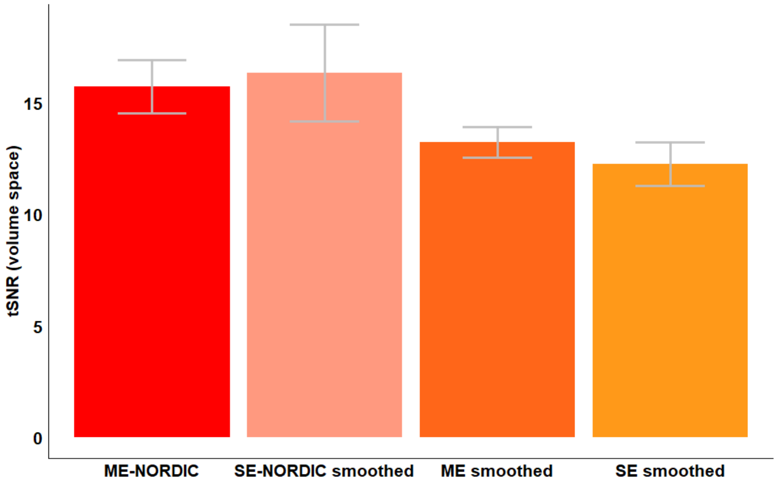

Figure S6: Experiment on the impact of smoothing on tSNR results. Average tSNR across runs for PA001 calculated in volume space using FSL tools. Error bars represent SD across runs. ME, SE and SE-NORDIC data are smoothed to the same FWHM as ME-NORDIC data using 3dBlurToFWHM in AFNI (Average FWHM for all ~ 3.7 (ME smoothed =3.69, SE smoothed=3.69, SE-NORDIC smoothed = 3.72, ME-NORDIC=3.7). Additional smoothing increases similarity between SE and ME data in terms of tSNR, however the boost with NORDIC shown in Figure 1 (in CIFTI space) remains.

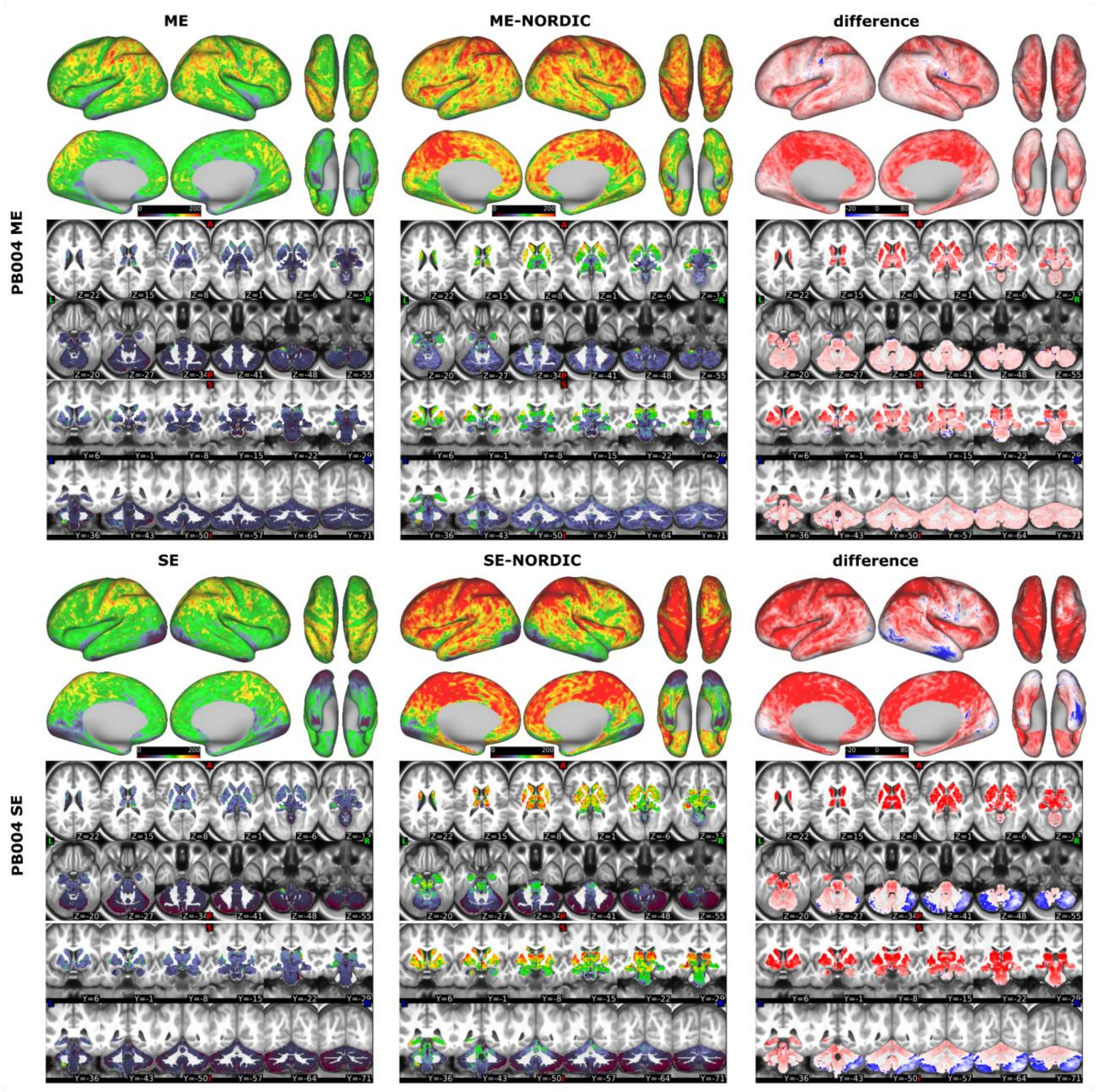

Figure S7: increase of tSNR with NORDIC for ME and SE data from PB004 (tSNR represents average of runs with >90% low motion).

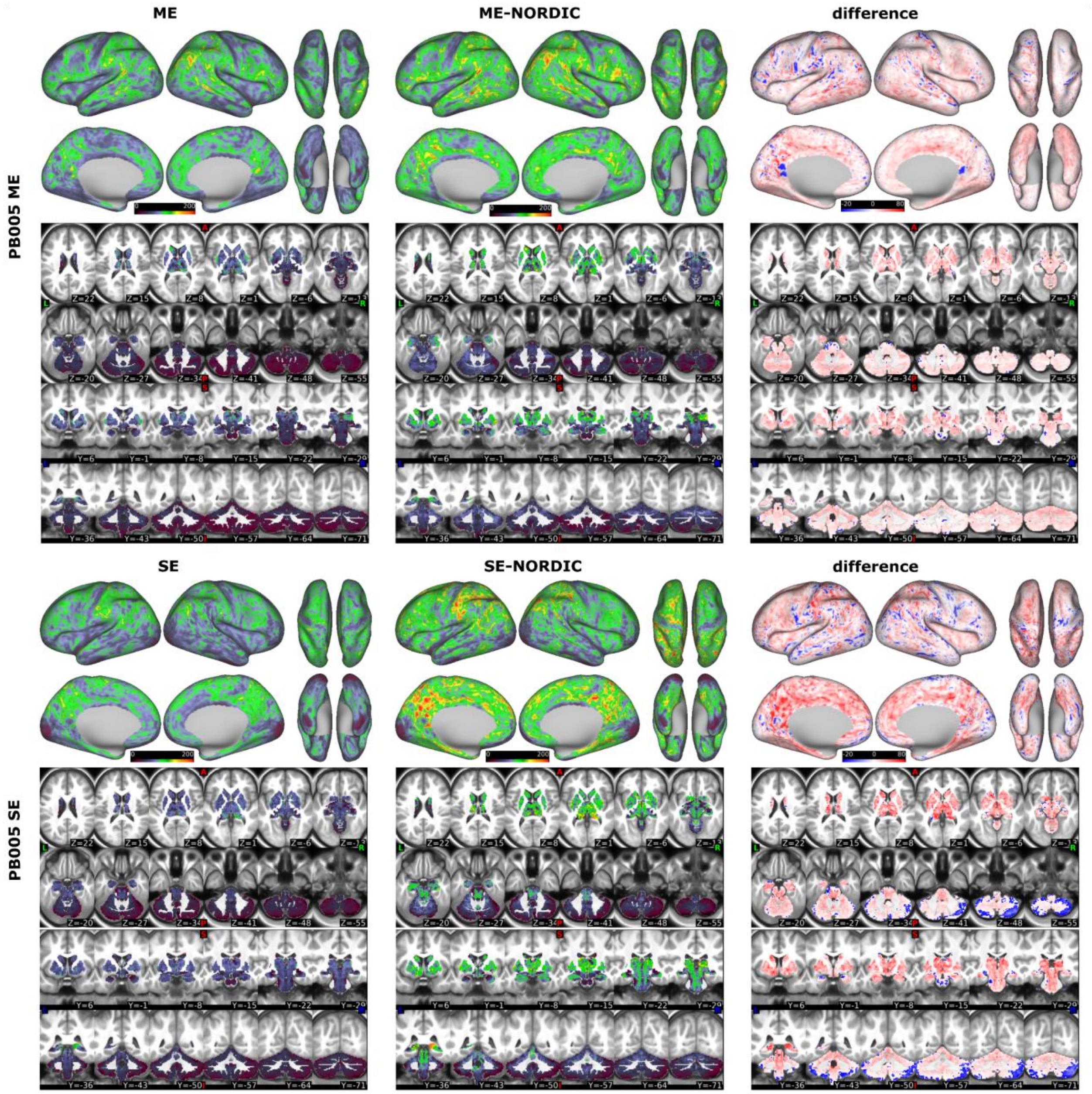

Figure S8: increase of tSNR with NORDIC for ME and SE data from PB005 (tSNR represents average of runs with >90% low motion).

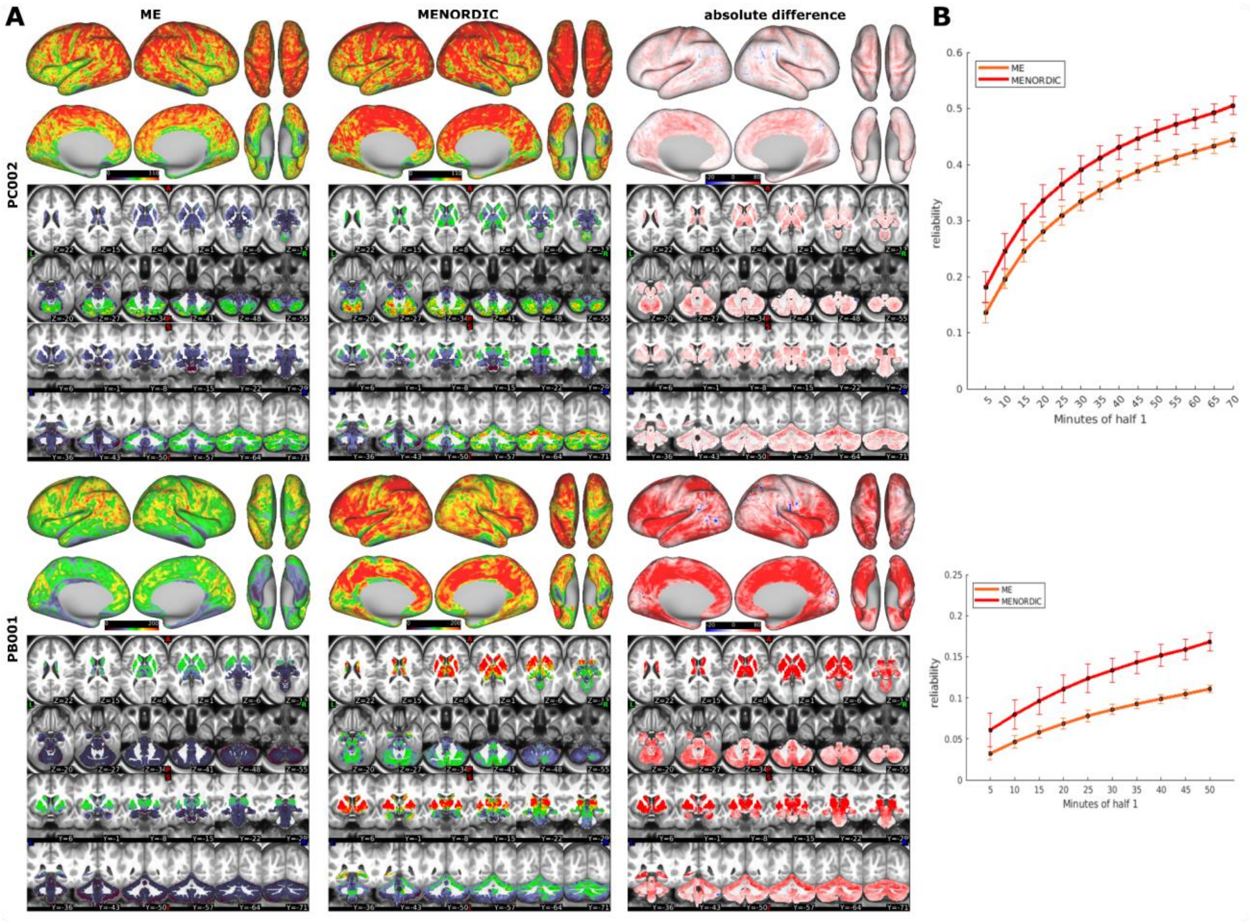

Figure S9: increase of tSNR (A) and reliability (B) with NORDIC for ME data from PC002 and PB001 (tSNR represents average of runs with >90% low motion). Curves represent the average reliability across all grayordinates and 100 permutations of the run order. Error bars show SD across permutations.

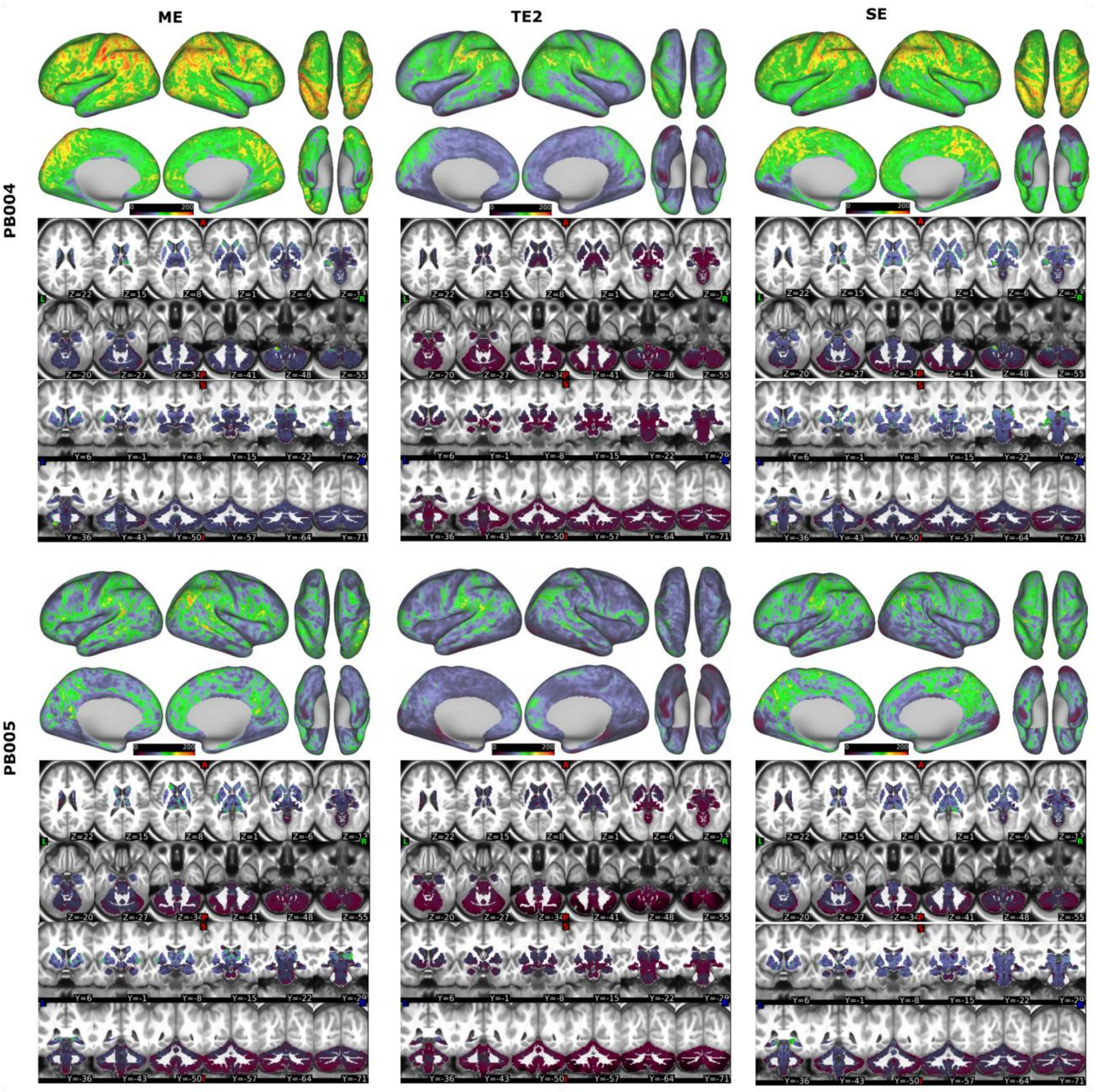

Figure S10: tSNR comparison between ME, SE and TE2 (second echo of ME data only). This comparison addresses whether the differences in TR or flip-angle between ME and SE account for some of the improvements in tSNR seen for ME. PB004 tSNR for ME is highest (M = 95.4, SD = 38.33) compared to TE2 (M = 64.44, SD = 31.98) or SE (M = 90.2, SD = 38). PB005 tSNR for ME is highest (M = 68.92 , SD = 27.5) compared to TE2 (M = 51.91, SD = 26.04) or SE (M = 68.24, SD = 26.94). TE2 shows no improvements over SE but rather a decrease in tSNR.

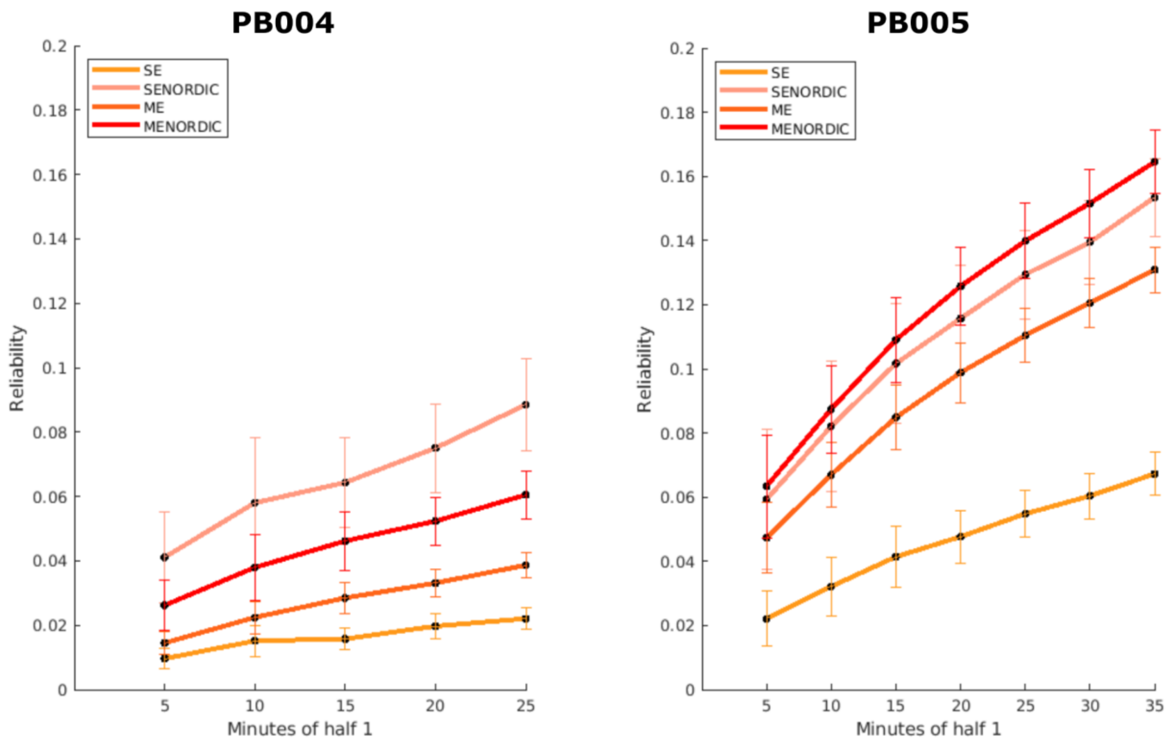

Figure S11: Increase of reliability with NORDIC for ME and SE data. Curves represent the average reliability across all grayordinates and 100 permutations of the run order. Error bars show SD across permutations.

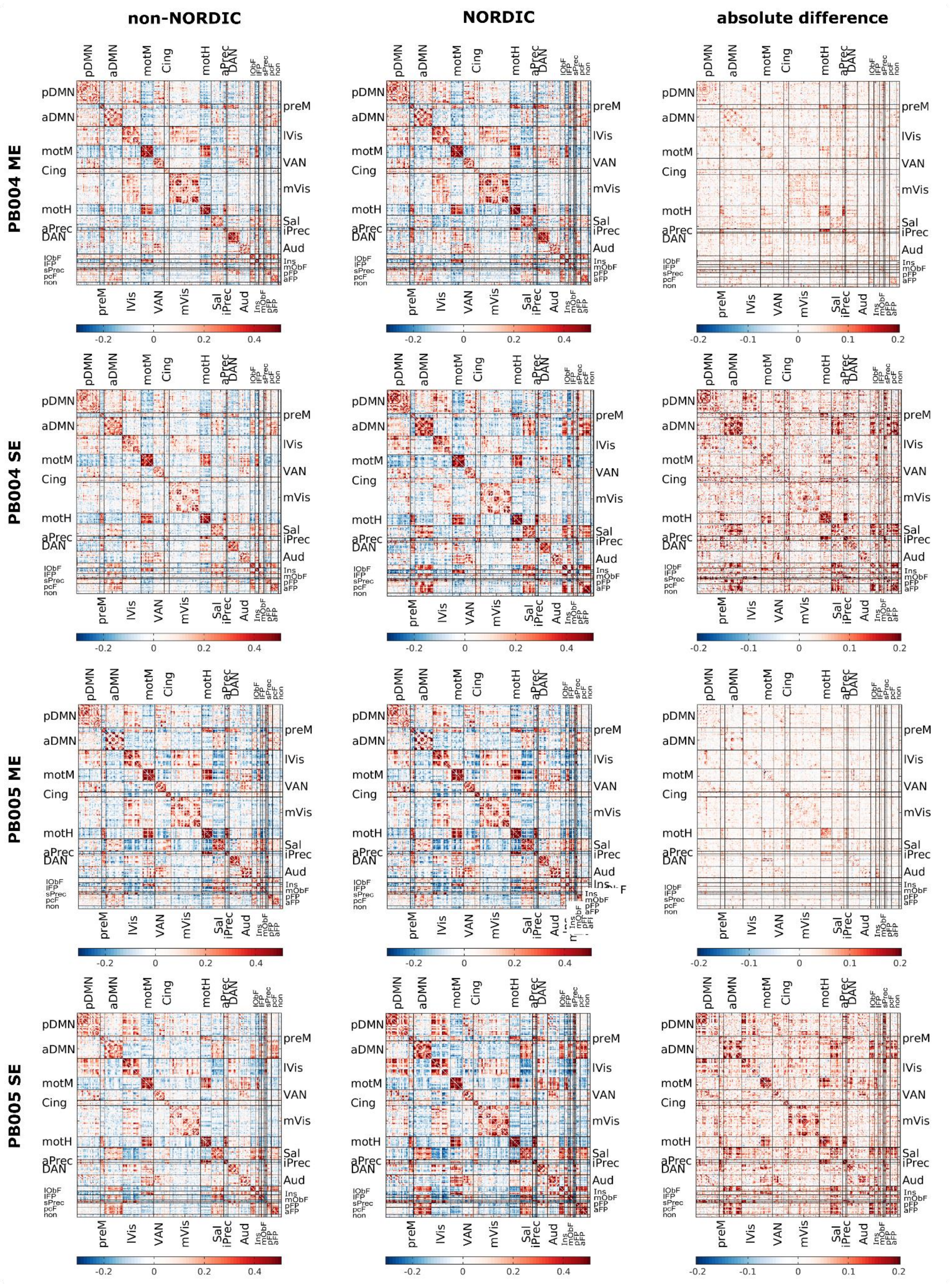

Figure S12: Connectivity matrices from parcellated time series (baby specific parcels) showing the increase of connectivity strength with NORDIC for both ME and SE data in PB004 and PB005.

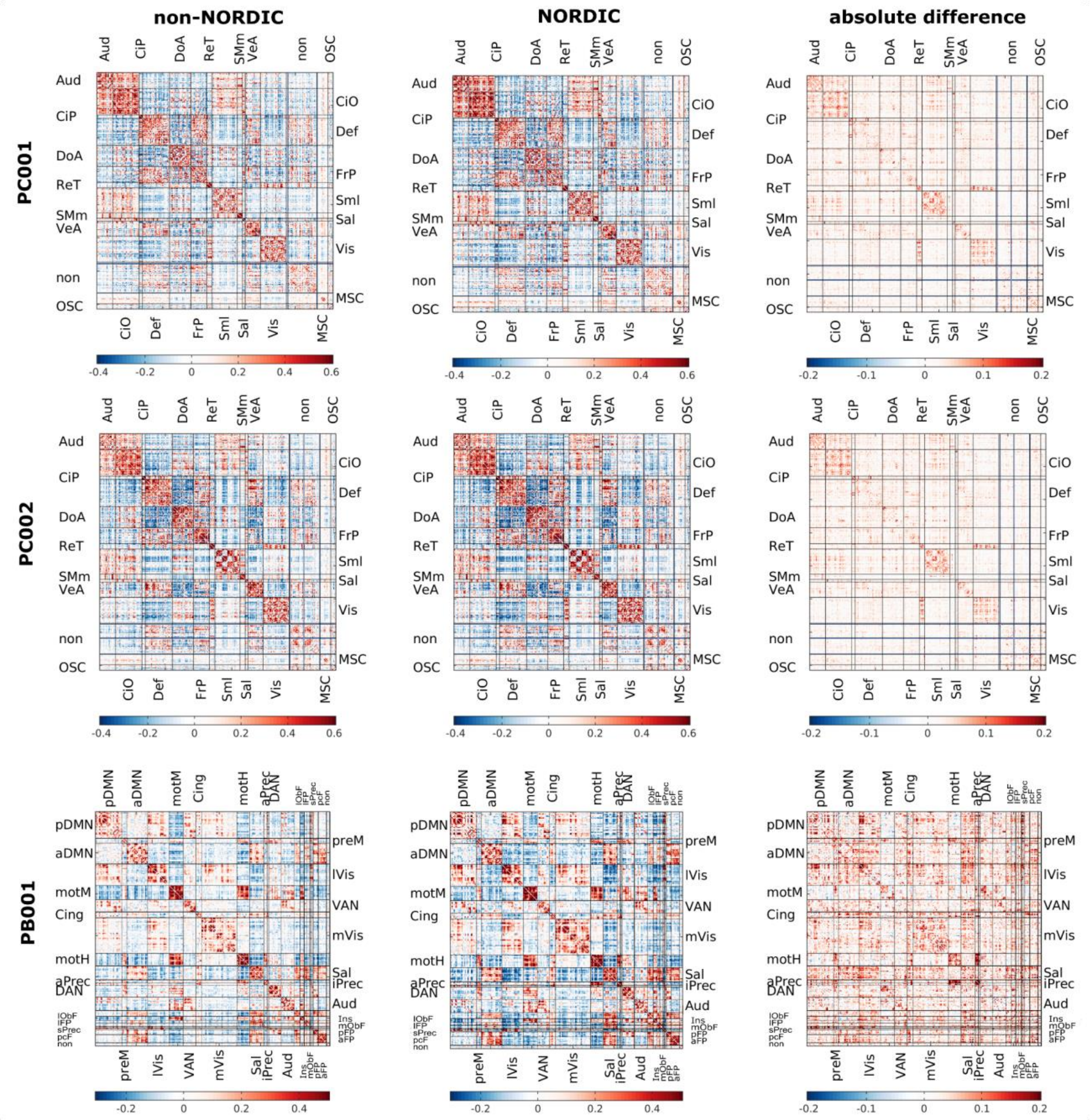

Figure S13: Connectivity matrices from parcellated time series (Gordon parcels for PC subjects and baby specific parcels for PB subject) showing the increase of connectivity strength with NORDIC for ME data from developmental populations.

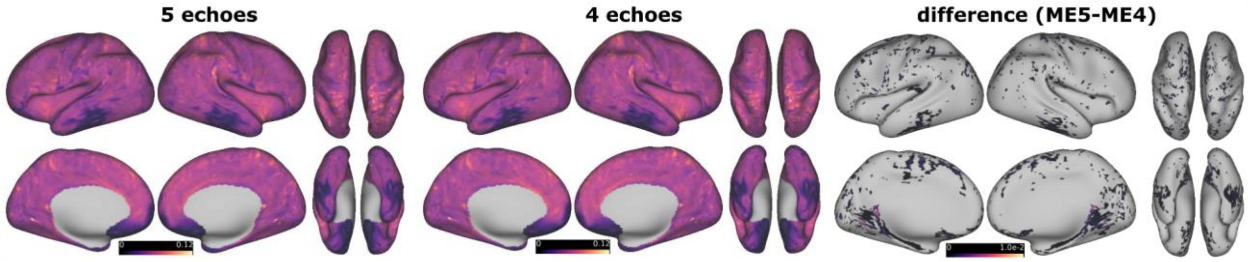

Figure S14: T2 values (in seconds) for PA001 calculated from either all 5 echoes or the first 4 echoes, showing that using a 4 echo sequence does not impact estimation of T2* values (average of runs with >90% low motion).*

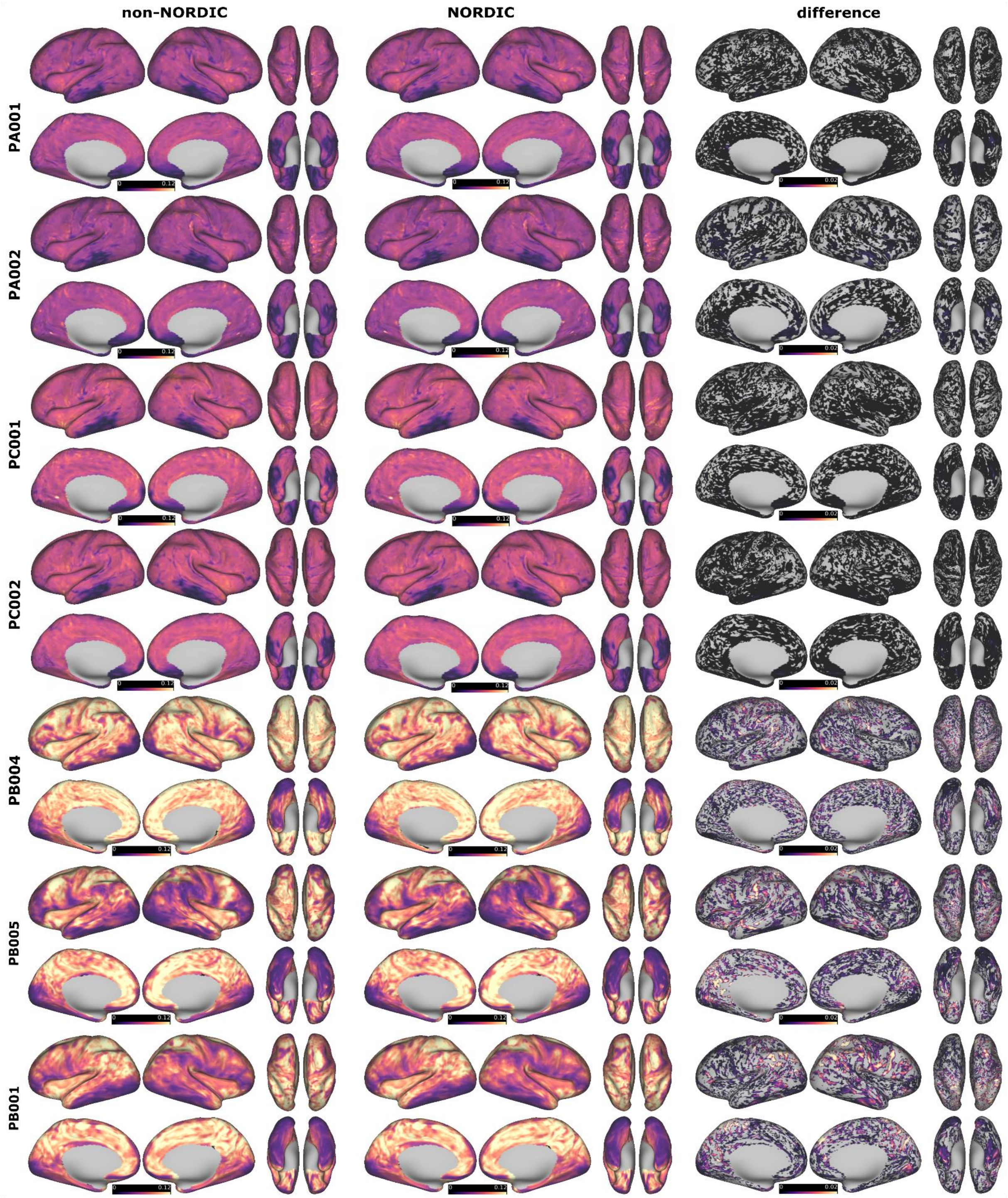

Suppl Figure 15: T2 values (in seconds) for all precision imaging subjects for data with and without NORDIC denoising prior to echo combination (average of runs with >90% low motion). NORDIC denoising only marginally changes T2* estimates.*

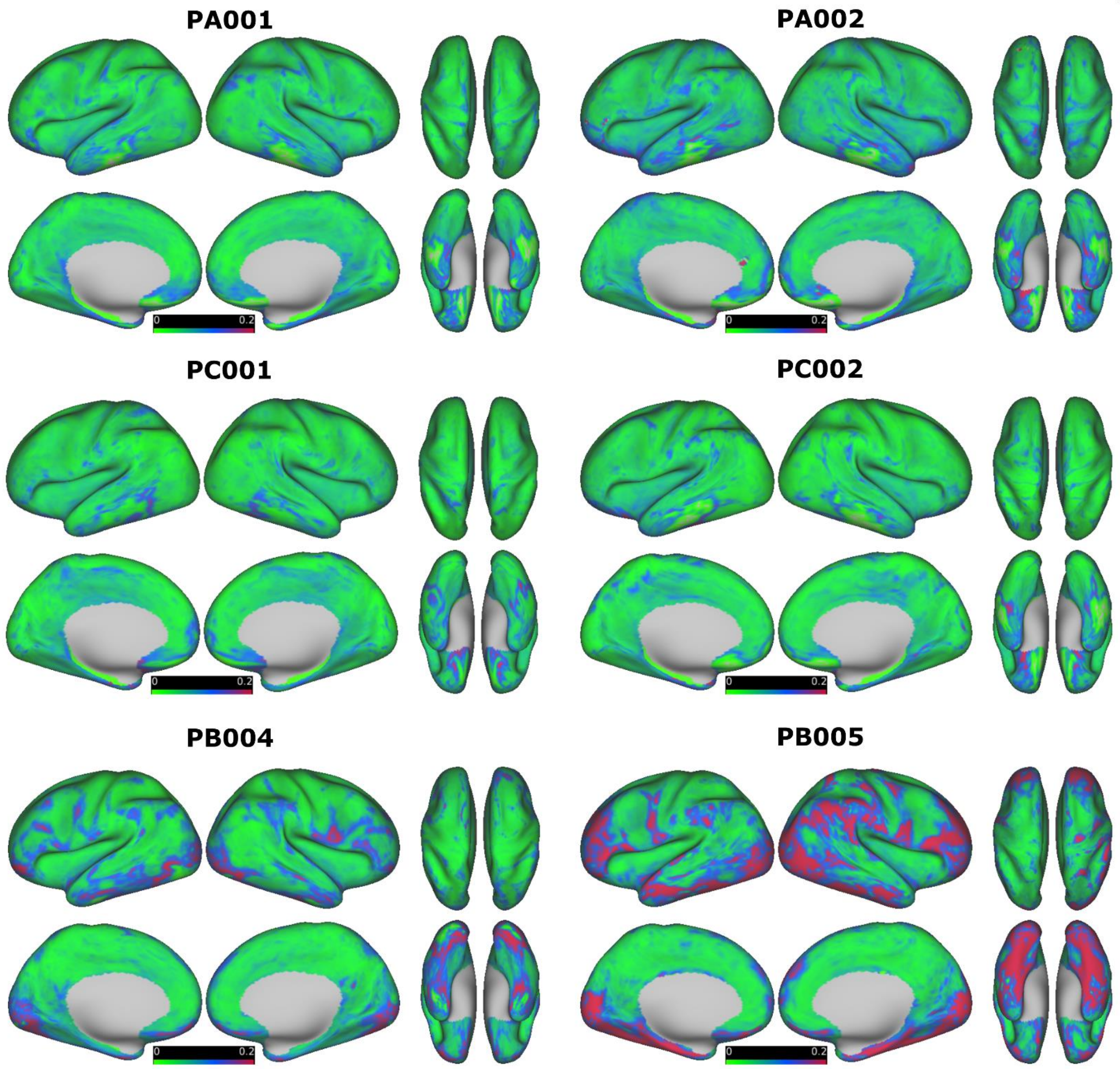

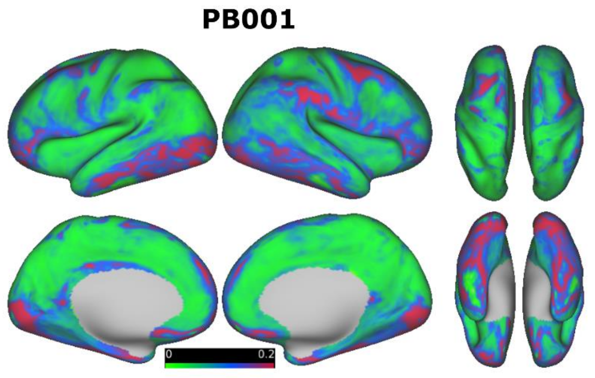

	mean	median	std	5th pct	95th pct
**PA001**	0.055	0.05	0.023	0.031	0.101
**PA002**	0.066	0.06	0.037	0.037	0.122
**PC001**	0.053	0.048	0.023	0.028	0.097
**PC002**	0.052	0.047	0.025	0.026	0.099
**PB004**	0.067	0.053	0.038	0.032	0.148
**PB005**	0.096	0.069	0.065	0.036	0.226
**PB001**	0.081	0.063	0.05	0.033	0.182

Figure S16: T2 and S0 model fit quantified from the root mean squared error (RMSE) output by Tedana version 24.0.1, normalized and projected to surface space. RMSE values were normalized by dividing them by the mean signal across echoes across a given run.*

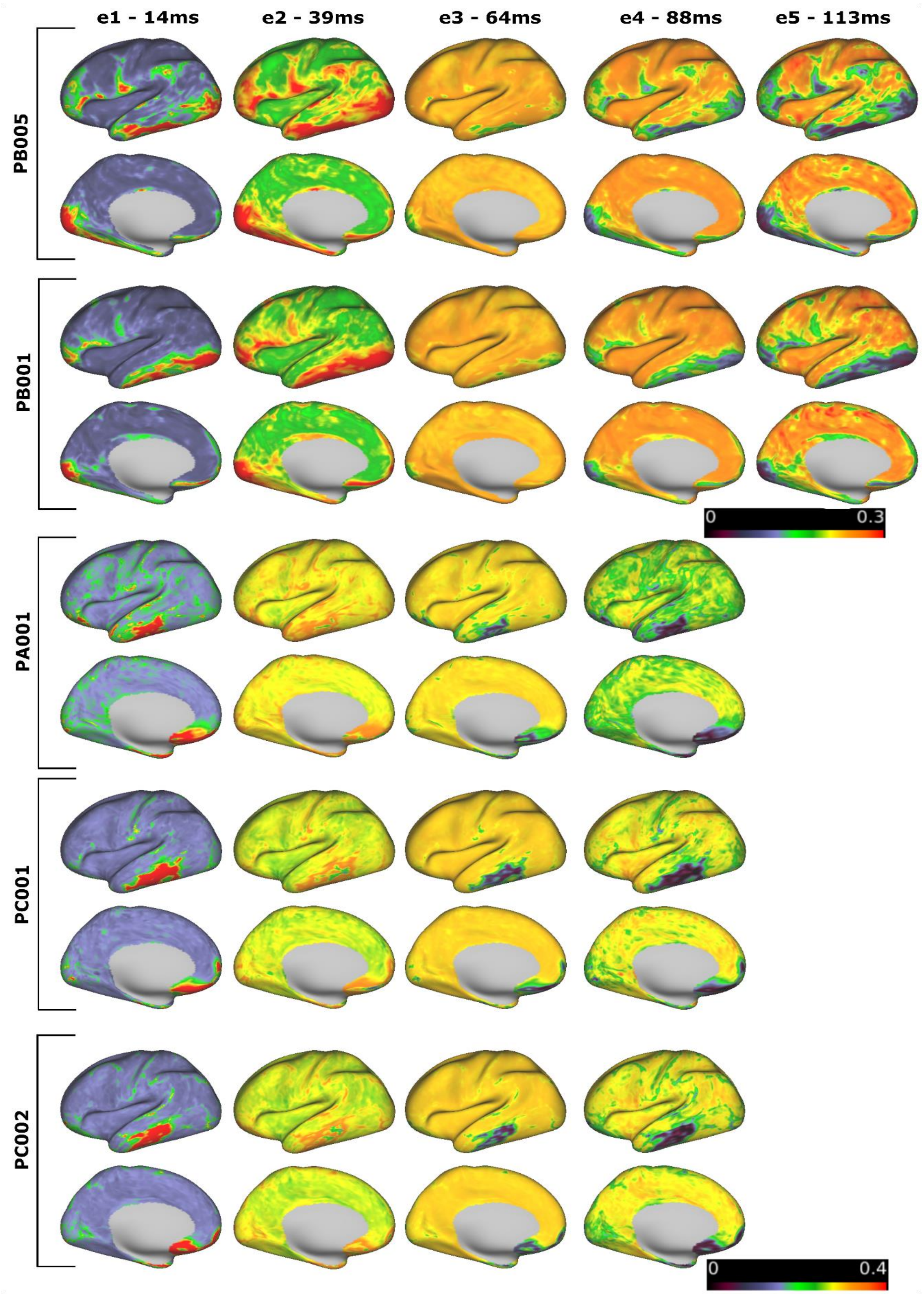

Figure S17: T2 based echo weighting for all precision imaging subjects not depicted in Figure 5. PA001, PC001 and PC002 were acquired with a 4 echo sequence. Despite lacking the last echo, their weighting scheme very much resembles PA001 shown in Figure 5.*

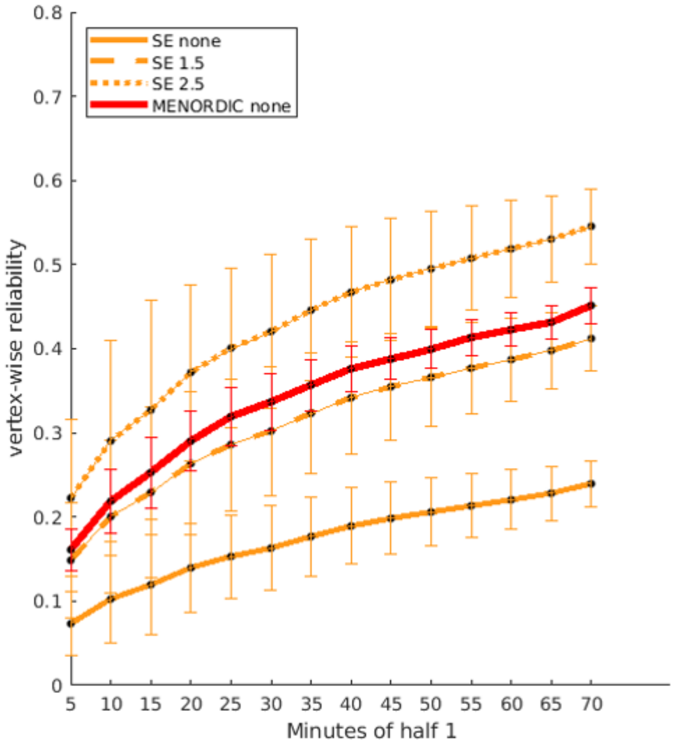

Figure S18: Variations in reliability with variations in data smoothing (SE and ME data PA001). Curves represent the average reliability across all grayordinates and 100 permutations of the run order. Error bars show SD across permutations. SE data were smoothed with a smoothing kernel of sigma = 1.5 mm and 2.5 mm respectively.

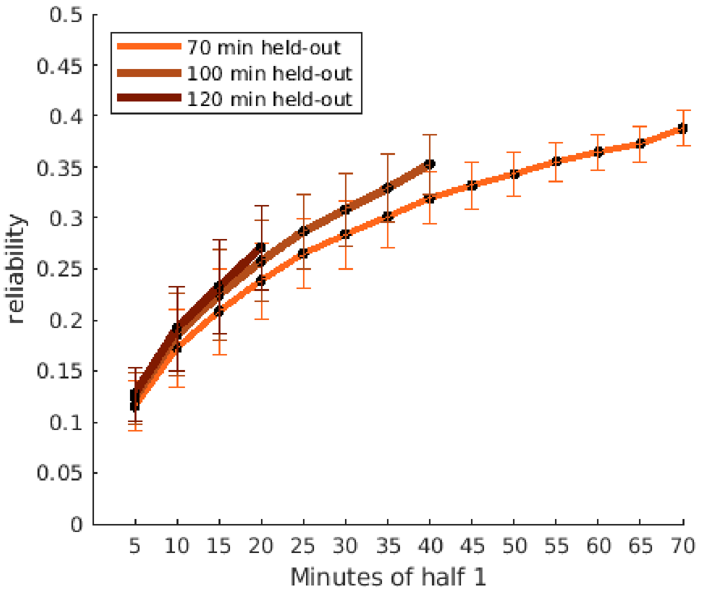

Figure S19: Variations in reliability with variations in held out data used for calculation (ME data PA001). Curves represent the average reliability across all grayordinates and 100 permutations of the run order. Error bars show SD across permutations.

	ME				SE			
	**Run**	**#frames**	**#good**	**percent**	**Run**	**#frames**	**#good**	**percent**
PA001	1	547	547	100	1	1124	1106	98.4
	2	547	547	100	2	1124	1103	98.13
	3	547	545	99.63	3	647	595	91.96
	4	547	545	99.63	4	1124	1090	96.98
	5	547	545	99.63	5	1124	1078	95.91
	6	547	546	99.82	6	1124	996	88.61
	7	547	541	98.9	7	1124	1041	92.62
	8	547	546	99.82	8	1124	1051	93.51
	9	547	547	100	9	597	493	82.58
	10	547	543	99.27	10	1124	1043	92.79
					11	597	495	82.91
					12	1124	1079	96
		**160.54 min**	**160.02 min**	**M=99.97**		**159.43 min**	**148.93 min**	**M=92.53**
PA002	1	510	506	99.22	1	1124	1111	98.84
	2	510	508	99.61	2	1124	1109	98.67
	3	441	416	94.33	3	1124	1115	99.2
	4	441	426	96.6	4	1124	1110	98.75
	5	441	430	97.51	5	1124	1107	98.49
	6	510	510	100	6	1124	582	51.78
	7	510	508	99.61	7	1124	1101	97.95
	8	510	510	100	8	1124	1097	97.6
	9	510	509	99.8	9	1124	1115	99.2
	10	510	506	99.22	10	1124	1098	97.69
	11	510	510	100				
	12	510	510	100				
	13	510	510	100				
	14	510	510	100				
		**203.48 min**	**201.6 min**	**M=98.99**		**149.87 min**	**140.6 min**	**M=93.82**
PC001	1	547	547	100				
	2	547	547	100				
	3	337	332	98.52				
	4	547	541	98.9				
	5	547	546	99.82				
	6	337	335	99.41				
	7	547	547	100				
	8	547	527	96.34				
	9	337	317	94.06				
	10	547	538	98.35				
	11	547	545	99.63				
	12	337	337	100				
		**168 min**	**166.1 min**	**M=98.75**				
PC002	1	547	541	98.9				
	2	547	544	99.45				

	3	337	337	100				
	4	547	541	98.9				
	5	547	546	99.82				
	6	337	335	99.41				
	7	547	513	93.78				
	8	337	326	96.74				
	9	547	508	92.87				
	10	547	536	97.99				
	11	337	326	96.74				
		**151.95 min**	**148.91 min**	**M=97.69**				
PB004	1	317	305	96.21	1	420	412	98.1
	2	317	301	94.95	2	131	131	100
	3	317	209	65.93	3	4420	296	70.48
	4	317	198	62.46	4	420	401	95.48
	5	317	167	52.68	5	420	416	99.05
	6	317	304	95.9	6	420	415	98.81
	7	130	126	96.92	7	420	413	98.33
	8	110	104	94.55	8	420	142	98.1
	9	317	293	75.39				
		**72.17 min**	**57.32 min**	**M=81.67**		**77.29 min**	**72.88 min**	**M=94.79**
PB005	1	317	148	46.69	1	420	380	90.48
	2	317	177	55.84	2	420	356	84.76
	3	181	134	74.03	3	420	286	68.1
	4	317	272	85.80	4	420	172	40.95
	5	317	279	88.01	5	284	94	33.1
	6	317	149	47	6	420	399	95
	7	317	162	51.1	7	420	321	76.43
	8	317	292	92.11	8	115	61	53.04
	9	317	310	97.79	9	301	198	65.78
	10	317	209	65.93	10	420	337	80.24
	11	317	173	54.57	11	420	267	63.57
	12	317	258	81.39	12	420	293	69.76
	13	317	103	32.49	13	420	227	54.05
		**116.96 min**	**78.25 min**	**M=67.14**		**123.32 min**	**85.34 min**	**M=67.33**
PB001	1	230	226	98.26				
	2	230	227	98.7				
	3	230	135	58.7				
	4	230	77	33.48				
	5	230	217	94.35				
	6	230	227	98.7				
	7	230	110	47.83				
	8	230	226	98.26				
	9	230	223	96.96				
	10	230	225	97.83				
	11	230	196	85.22				
	12	230	67	29.13				

	13	230	227	98.7				
	14	230	227	98.7				
	15	230	226	98.26				
	16	230	146	52.17				
	17	230	120	80.87				
	18	230	186	80.88				
	19	230	207	90				
	20	230	221	96.09				
	21	230	170	73.91				
		**141.76 min**	**112.09 min**	**M=83.02**				

Supplementary Table 1: Frames per run and frames per run remaining after motion censoring. Infants: FD<0.3, adults FD<0.2; gray: excluded as below 30% of the run.

BOLD sequence	Subjects	TE	TR	MB factor	Flip angle	Resolution
ME	adults/children /infants	14.2ms, 38.93ms, 63.66ms, 88.39ms, 113.12ms	1.761s	6	68°	2mm
SE	adults	37ms	0.8s	8	52°	2mm
SE	infants	37ms	1.51s	4	52°	2mm

Supplementary Table 2: Overview of BOLD sequences used in this study

	amount of data		non-NORDIC	NORDIC	absolute difference
PA001	140 min	ME	M =0.172, SD =0.407	M =0.193, SD =0.411	M =0.028, SD = 0.026
			min=-0.765, max= 1.955	min=-0.784, max= 1.988	min=-0.087, max= 0.25
		SE	M =0.16, SD =0.403	M =0.178, SD =0.407	M =0.028, SD =0.025
			min=-0.733, max= 1.767	min=-0.758, max= 1.756	min=-0.705, max= 0.255
PA002	140 min	ME	M =0.179, SD =0.405	M =0.211, SD = 0.413	M =0.042, SD = 0.04
			min=-0.69, max= 1.61	min=-0.748, max= 1.716	min=-0.328, max= 0.388
		SE	M =0.18, SD =0.407	M =0.213, SD =0.416	M =0.04, SD =0.04
			min=-0.805, max= 1.527	min=-0.84, max= 1.543	min=-0.123, max= 0.307
PC001	160 min	ME	M =0.156, SD = 0.401	M =0.172, SD = 0.405	M =0.022, SD = 0.022
			min= -0.544, max= 1.575	min=-0.569, max= 1.573	min=-0.107, max= 0.285

PC002	140 min	ME	M =0.161, SD =0.404	M =0.176, SD = 0.408	M =0.022, SD = 0.02
			min=-0.577, max= 1.653	min=-0.598, max= 1.684	min=-0.1, max= 0.247
PB004	55 min	ME	M = 0.122, SD = 0.436	M = 0.135, SD = 0.438	M = 0.029, SD = 0.025
			min=-0.346, max=1.276	min=-0.377, max=1.391	min=-0.136, max=0.261
		SE	M =0.115, SD =0.434	M =0.141, SD =0.441	M =0.051, SD =0.052
			min=-0.331, max= 1.155	min=-0.405, max= 1.497	min=-0.235, max= 0.581
PB005	75 min	ME	M = 0.128, SD = 0.438	M = 0.14, SD = 0.44	M = 0.024, SD = 0.022
			min=-0.455, max=1.395	min=-0.493, max=1.45	min=-0.197, max=0.262
		SE	M =0.12, SD =0.435	M =0.151, SD =0.442	M =0.049, SD =0.048
			min=-0.352, max= 1.078	min=-0.418, max= 1.646	min=-0.203, max= 0.599
PB001	110 min	ME	M =0.121, SD = 0.437	M =0.146, SD = 0.44	M = 0.044, SD = 0.04
			min=-0.34, max=1.376	min=-0.428, max=1.427	min=-0.302, max=0.406

Supplementary Table 3: overall connectivity strength of parcellated data with and without NORDIC for precision imaging participants. All fisher z-transformed values, range excludes the matrix diagonal.

		non-NORDIC	non-NORDIC >90% low motion runs only	NORDIC	NORDIC >90% low motion runs only
PA001	ME	3.47	3.47	3.7	3.7
	SE	2.73	2.73	2.8	2.8
PA002	ME	3.26	3.26	3.35	3.35
	SE	2.5	2.5	2.51	2.51
PC001	ME	3.25	3.25	3.43	3.43
PC002	ME	3.39	3.39	3.61	3.61
PB004	ME	7.02	6.8	7.41	7.25
	SE	6.28	6.26	6.6	6.58
PB005	ME	8.48	7.89	9.13	8.53

	SE	7.25	6.97	7.69	7.46
PB001	ME	7.73	7.37	8.5	8.31

Supplementary Table 4: Data smoothness estimated by full width half max (FWHM) for all participants. Table lists mean across all runs and all runs with >90% low motion data. Note that all data are in MNI space, which increases smoothing for infant data compared to children and adults.

		Percent change in tSNR with NORDIC within mask	Percent change in tSNR with NORDIC outside mask
PA001	ME	18.94 %	25.43 %
	SE	10.11 %	37.51 %
PA002	ME	5.29 %	16.95 %
	SE	0.39 %	25.07 %
PB004	ME	17.1 %	34.42 %
	SE	7.8 %	50.97 %
PB005	ME	17.94 %	18.34 %
	SE	11.81 %	23.19 %

Supplementary Table 5: Exemplary analysis to demonstrate spatial specificity of improvements of tSNR with NORDIC between SE and ME. Mask is defined as regions with lowest 5 % of signal, representing regions with high signal dropout in SE data. Example in PA001 is depicted in Figure S2. Outside these regions, SE data benefits more from NORDIC denoising. Inside those regions, ME data benefits more from NORDIC denoising, as more signal is recovered through optimal echo combination.
